# Review of Fundamental Active Current Extraction Techniques for SAPF

**DOI:** 10.3390/s22207985

**Published:** 2022-10-19

**Authors:** Jan Baros, Vojtech Sotola, Petr Bilik, Radek Martinek, Rene Jaros, Lukas Danys, Petr Simonik

**Affiliations:** 1 Department of Cybernetics and Biomedical Engineering, VSB–Technical University of Ostrava, 17. listopadu 15, 708 33 Ostrava, Czech Republic; 2 Department of Electronics, VSB–Technical University of Ostrava, 17. listopadu 15, 708 33 Ostrava, Czech Republic

**Keywords:** active filtration, harmonic extraction, shunt active power filter, synchronization, total harmonic distortion

## Abstract

The field of advanced digital signal processing methods is one of the fastest developing scientific and technical disciplines, and is important in the field of Shunt Active Power Filter control methods. Shunt active power filters are highly desirable to minimize losses due to the increase in the number of nonlinear loads (deformed power). Currently, there is rapid development in new adaptive, non-adaptive, and especially hybrid methods of digital signal processing. Nowadays, modern methods of digital signal processing maintain a key role in research and industrial applications. Many of the best practices that have been used to control shunt active power in industrial practice for decades are now being surpassed in favor of new progressive approaches. This systematic research review classifies the importance of using advanced signal processing methods in the field of shunt active power filter control methods and summarizes the extant harmonic extraction methods, from the conventional approach to new progressive methods using genetic algorithms, artificial intelligence, and machine learning. Synchronization techniques are described and compared as well.

## 1. Introduction

The development of semiconductor technology has led to the massive expansion of electronics. These electronic devices are directly connected to the electrical grid, where they can cause problems because of their nonlinear load characteristic. This nonlinearity causes harmonic distortion of voltage or current, which leads primarily to economic losses and secondarily to excessive electrical network overloads [1,2].

These problems with higher harmonic components can be solved in two ways, via passive or active filtration. Passive filtration methods can be a suitable choice for isolating transformer connections (e.g., Delta-Star with lead out neutral conductor (Dyn) connection suppresses the third harmonic [3,4]), as can connecting a resonant filter [5,6]. Passive filters have been widely used due to their low cost and significant efficiency in suppressing harmonics. Unfortunately, these filters have multiple disadvantages. For example, if the load is a controlled rectifier, it is necessary to compensate only the significant odd harmonic components (third and fifth). In this case, the filter is optimized. However, if the load dynamically changes its harmonic spectrum, the filter becomes inefficient. Another disadvantage is that filtering characteristics are significantly influenced by the impedance of the electrical network. With an unsuitable design, it is possible to cause a serial or parallel resonance on the distribution network side. Another negative aspect of passive filtration is that due to high-power flows it is necessary to design the components appropriately. This is reflected in their size, as such filters take up a lot of physical space in substations. Furthermore, it is necessary to consider that filters at lower frequencies have a capacitance character, and therefore compensate the power factor.

As stated above, another option is to use active filtration [7,8], which has become relevant only due to the development of switchable semiconductor components such as Gate turn-off (GTO), Integrated Gate Commutated Thyristors (IGCT), and especially Insulated Gate Bipolar Transistors (IGBT). In addition to these components, development has been accelerated by the massive growth in the computing power of microcomputers on which it is possible to implement various complex computing methods. Active filters, thanks to their functionality, are able to bypass the disadvantages of passive filters. First of all, it is possible to react dynamically to changes in the conditions of the electrical network or in the frequency harmonic spectrum of the connected load. Thus, there is no need to design a filter for each harmonic component separately, nor is there a need to design a filter for any specific load. Unfortunately, the advantages of active filters are countered by several significant disadvantages. Due to the cost of power semiconductor switching elements, rectifier diodes, sensors, and capacitors, the initial purchase and maintenance costs are very high. High demands are placed on the computing unit as well, which is the “brain” of the device. The task of this unit is to calculate all mathematical operations and to control the generation of the compensation current signal. In addition, the response time of this control system must ideally be instantaneous, or at least minimal. Another negative aspect of active filtration is the need to produce a de facto duplicate of the current taken with the opposite sign and without the fundamental harmonic component. Thus, while power losses are not undesirable in terms of functionality, in terms of cost the losses are considerable, as all power outside the fundamental harmonic component must be produced with the opposite sign, i.e., duplicated.

Active filters can be divided into serial active filters, shunt active filters, or their combination. Serial active filters are used to solve voltage issues in the electrical network, such as voltage amplitude correction, compensation of voltage drops, removal of voltage harmonic components, overshoots, or power cuts (if powered from backup power source), thus achieving balanced voltage. This type of filter does not allow compensation of current harmonics. Shunt active filters are used to resolve ongoing issues in the electrical network, such as removal of current harmonic components and reactive power compensation. Both types of filters can be combined, with the serial part taking care of the voltage and the shunt part taking care of the current. Active filters can be combined with passive filters as well [9,10]. This combination is known as a hybrid filter. Such filters reduce the electromagnetic interference possible with active filters and prevent resonant interactions between the passive filter and the network, thus combining the advantages of both technologies.

Currently, active filters are on the rise; unfortunately, at present this development mainly concerns academia. However, stagnation can be observed in the electrical power industry, where proven yet obsolete solutions are used instead of integrating new technologies. The electrical power industry is not in favour of innovation, mainly for economic reasons; the deployment of new and more advanced equipment on a mass scale is likely to require a significant increase in costs, and thus a reduction in profits. Another aspect is component failure, as each sensor and switching element has a limited service life and its replacement or repair increases costs. The current trend in the academic research environment is to design methods that can work with as low a number of sensors as possible, e.g., measuring only two currents [11,12] instead of three voltages and three currents. Therefore, instead of six sensors, only two sensors are used and the remaining variables are calculated or estimated (see adaptive methods). As the number of sensors decreases, both the purchase price and the service price decrease. However, this trend is not followed in industry; on the contrary, it is economically efficient to produce and use obsolete solutions that have a declared functionality through tens of years of deployment. A parallel scenario arises in other electrical power fields that do not directly deal with current harmonics compensation.

This review aims to present classical, modern, and hybrid control methods in various aspects of Shunt-Active Power Filter (SAPF) control. These control methods can be further divided into two groups: 1. Harmonic Extraction Algorithms [13,14,15,16,17,18,19,20,21,22,23,24,25]; and 2. Synchronization Techniques [26,27,28,29,30,31,32,33,34,35,36,37,38,39,40,41,42,43,44,45,46,47,48,49,50].

**Figure 1:**
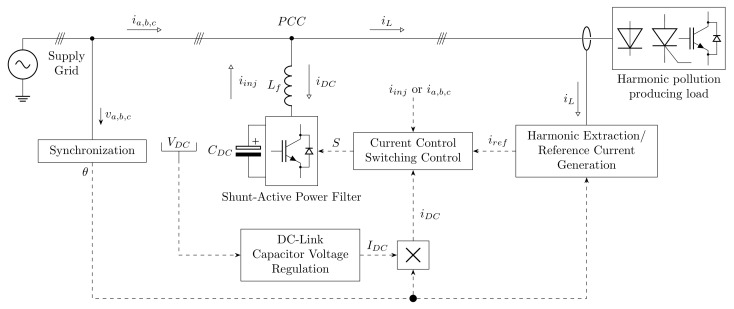
Simplified block diagram and working principle of SAPF.

This SAPF architecture can be seen in the simplified diagram shown in Figure 1. All control algorithms are implemented on a common powerful control unit (a Field Programmable Gate Array (FPGA) or Digital Signal Processor (DSP)) which is able to dynamically respond to the occurrence of higher harmonics in the distribution network, and thus to compensate these higher harmonics in real time or with minimal delay. It is worth mentioning that the power inverter itself is a source of harmonics; thus, the inverter is connected to the network using a low-pass LC filter. This LC filter is used to reduce the higher harmonic components generated by SAPF switching pulses.

The task of the Harmonic Extraction Algorithm (often called the Reference Current Generation Algorithm in the literature) is to generate a reference current that corresponds to the distortion contained in a harmonically polluted distribution network. It separates the fundamental i(n) and higher $\sum i_{h}\left(n\right)$ harmonic components contained in the current taken by the load $i_{L}\left(n\right)$. The desired reference current $i_{ref}\left(n\right)$ is then equal to the negation of higher harmonic components; thus, the label is consequently changed to $i_{inj}\left(n\right)$. The sum of $i_{ref}\left(n\right)$ and $i_{L}\left(n\right)$ is used for compensation, and only the fundamental component i(n) remains in the source current signal. The procedure described above can be seen in the following Equations (1)–(3):

(1) $$\begin{array}{c} i_{L}\left(n\right)=i\left(n\right)+\sum i_{h}\left(n\right) \end{array}$$

(2) $$\begin{array}{c} i_{inj}\left(n\right)=-\sum i_{h}\left(n\right) \end{array}$$

(3) $$\begin{array}{c} i_{ref}\left(n\right)=i\left(n\right)-i_{L}\left(n\right). \end{array}$$

The main requirements of filter methods are accuracy and speed of current generation, where the generated reference current must be generated as accurately as possible within the shortest possible period. Other requirements are the speed of convergence, stability, minimal mean square error rate, and number of floating point operations in one iteration. Algorithms can be divided into three basic groups: time domain control [13,14,15,16,18,19,20,22], frequency domain control [21,51], and adaptive methods [23,24,25]. These methods are described in greater detail in Section 2.

Another important element of SAPF control is the synchronization algorithm, which has the task of supplying information about the current phase θ(t) of the source voltage in order for the reference compensation current to be generated correctly in phase. As mentioned earlier, compensation is performed using the sum of two current signals. In order for the sum to be correct, the current signals must be in exactly the same phase. As seen above, obtaining information on the current phase and accuracy demands very low latency. Most methods are based on Phase-Locked Loop (PLL) synchronizers, although unconventional methods can be encountered; see Section 3 on synchronization methods.

Other algorithms required for SAPF address DC-link Capacitor Voltage Regulation and Current Control. DC-link voltage control algorithms are necessary for the inverter to function properly; when the DC-link voltage is low, the inverter is unable to generate an injection current to compensate for higher harmonics. On the contrary, a higher value can cause the inverter to switch to pulse rectifier mode, which leads to an increase in DC-link voltage. This voltage increase can damage the capacitor or other electronic components of the inverter. Current control algorithms are used to generate an injection current that combines the requirements of a DC-link and a reference current at the same time. These methods can be divided into two basic groups, Direct Current Control (DCC) and Indirect Current Control (ICC), according to chosen type of feedback current. The control of power inverters is mainly realized using Pulse-Width Modulation (PWM), a comparative method, or polar Space Vector PWM (SVPWM). As these algorithms are de facto related to the hardware of the inverter instead of to computing software, they are not covered in this article. Relevant information about these methods can be found in [52,53,54,55,56,57].

In this publication, we introduce and discuss methods used at various stages of SAPF control for compensation of undesirable harmonic components. The following two sections deal with the individual SAPF control blocks, and every section presents the methods that are used for each. The sections pertaining to each method contain a brief description necessary to understand its principle along with several publications in which the method has been described, simulated, or implemented in real experiments. Every sections ends with a summary table where different methods are compared.

Here, it is important to mention that the comparison of individual methods is rather subjective, as the methods are necessarily implemented under varying conditions. Performing this “objective” comparison under identical conditions and quantifying “quality” (i.e., the dynamic response of different methods, their Total Harmonic Distortion (THD), and the ability to work under non-ideal conditions) of various methods is the future intention of the authors. The comparisons in this review are therefore intended to present the mentioned methods to the reader, and the tables are intended to provide a basic awareness of the advantages and disadvantages of various methods.

This publication ends with a thorough discussion of SAPF development and concludes with a brief summary of all the results herein.

## 2. Harmonic Extraction Algorithms

Basic control algorithms were originally designed for ideal voltage sources in which balanced and sinusoidal voltages can be assumed. However, this assumption is not met in real conditions; therefore, these methods have been either modified or newly developed to be able to work properly even under unbalanced and non-sinusoidal voltages. At present, harmonic extraction methods can be divided into three basic groups: control in the time domain [13,14,15,16,18,19,20,22], control in the frequency domain [21,51], and adaptive filtering [23,24,25]. This section describes methods from all three groups; in Table 1, a summary of the properties of these methods is provided.

### 2.1. Instantaneous Reactive Power Theory

This method (abbreviated as iPQ) works with instantaneous values in a three-phase system. This method is based on the Clark transform for converting a three-phase system *abc* into stationary αβ coordinates. In total, it is necessary to have three voltage sensors and three current sensors. Instantaneous active power p(t) and reactive power q(t) are calculated as follows:

(4) $$\begin{array}{c} p\left(t\right)=v_{\alpha }\left(t\right)\times i_{\alpha }\left(t\right)+v_{\beta }\left(t\right)\times i_{\beta }\left(t\right), \end{array}$$

(5) $$\begin{array}{c} q\left(t\right)=v_{\alpha }\left(t\right)\times i_{\beta }\left(t\right)+v_{\beta }\left(t\right)\times i_{\alpha }\left(t\right). \end{array}$$

**Figure 2:**
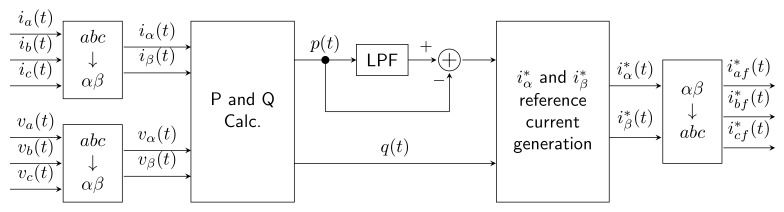
Block diagram of instantaneous reactive p-q theory.

The instantaneous active power p(t) is then divided into the fundamental active power part $\bar{p}\left(t\right)$ and the harmonic part $\tilde{p}\left(t\right)$. After this splitting, the fundamental part is neglected and the $\tilde{p}\left(t\right)$ part is used. Reference currents in the α and β coordinates are then back-calculated from $\tilde{p}\left(t\right)$ and q(t) and compensation currents are obtained by performing inverse Clark transformation. The whole procedure can be seen in Figure 2.

This method was originally proposed in 1984 by Akagi et al. [58] and has been gradually modified over many years [59,60,61,62,63,64,65]. Its advantages include easy implementation and, if the system is supplied with ideal voltage, excellent results under static conditions. Disadvantages include the need to measure three voltages and currents, and if the system is not supplied with ideal voltage, the results are highly distorted. This method is very sensitive to imbalances and high harmonic distortion in the voltage signals.

### 2.2. Cross-Vector Theory

**Figure 3:**
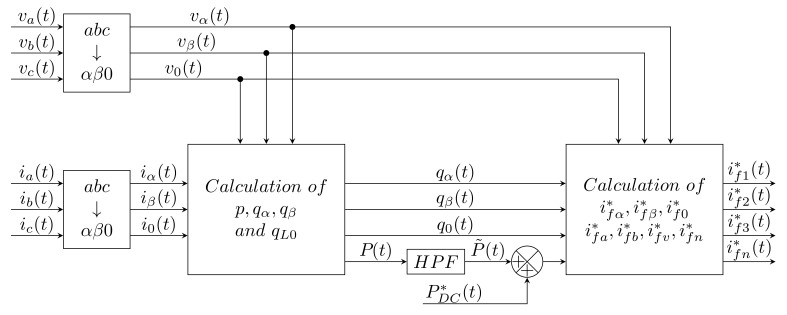
Diagram of Cross-Vector Theory.

A simple modification of the above method creates a new method called Cross-Vector Theory (CVT). Unlike iPQ, this method considers all components gained after Clark Transformation, extending the system to αβ0. This extension takes the the form of better suppression of higher harmonic components in the neutral conductor. For this reason, it is necessary to extend the overall system from a Three-Leg to a Four-Leg inverter. The block diagram of this method is shown in Figure 3.

In [18], the authors presented a comparison of the iPQ, PQR, and CVT theories. Their experiments were performed only in the MATLAB simulation environment on RC and RL loads. The results and conclusion show that while all methods are able to reduce the THD${}_{I}$ value to a low one, it does not fall below the 5% limit. Cross-Vector Theory appears to be the best in terms of having the smallest value of current flowing through the neutral wire. Furthermore, a modification of the Cross-Vector Theory method using PLL is mentioned which led to better results (about 3% THD${}_{I}$).

### 2.3. Rotating PQR Theory

The PQR method was designed to completely eliminate the zero-sequence current. This method uses two transformation processes. The first is the transformation of voltages and currents from abc coordinates to αβ0 coordinates [15,66,67]. The second transformation is the transformation of currents from αβ0 coordinates to pqr coordinates, which are rotated relative to the original fixed system by an ϕ angle. The method uses the following equation:

(6) $$\left[\begin{array}{c} i_{p}\\ i_{q}\\ i_{r} \end{array}\right]=\left[\begin{array}{ccc} \frac{v_{\alpha }}{v_{\alpha \beta 0}} & \frac{v_{\beta }}{v_{\alpha \beta 0}} & \frac{v_{0}}{v_{\alpha \beta 0}}\\ -\frac{v_{\beta }}{v_{\alpha \beta }} & \frac{v_{\alpha }}{v_{\alpha \beta }} & 0\\ -\frac{v_{0}v_{\alpha }}{v_{\alpha \beta 0}v_{\alpha \beta }} & -\frac{v_{0}v_{\beta }}{v_{\alpha \beta 0}v_{\alpha \beta }} & \frac{v_{\alpha \beta }}{v_{\alpha \beta 0}} \end{array}\right]\left[\begin{array}{c} i_{\alpha }\\ i_{\beta }\\ i_{0} \end{array}\right],$$

where $v_{\alpha \beta 0}=\;\sqrt{{v_{\alpha }}^{2}+{v_{\beta }}^{2}+{v_{0}}^{2}}$ and $v_{\alpha \beta }=\;\sqrt{{v_{\alpha }}^{2}+{v_{\beta }}^{2}}$ are the voltage vector magnitudes.

**Figure 4:**
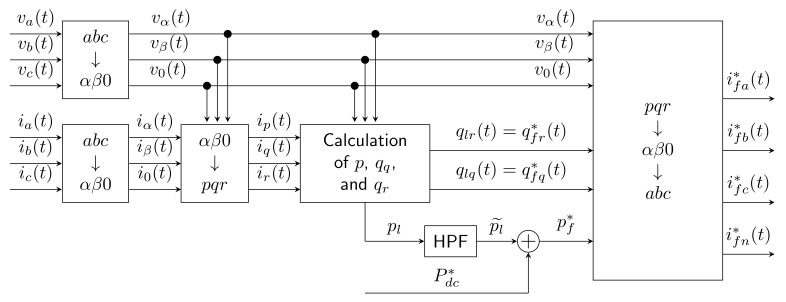
Block diagram of Rotating PQR Theory.

From these currents in *pqr* coordinates, the independent variables *p*, $q_{q}$, and $q_{r}$ are calculated. The last step is double inverse transformation from *pqr* to *abc* coordinates. See Figure 4 for a block diagram of Rotating PQR Theory.

Revuelta et al. [19] described various methods that were simulated and applied in three cases: sinusoidal balanced, sinusoidal unbalanced, and non-sinusoidal balanced source voltage. The PQR method was applied for a sinusoidal balanced source voltage, achieving zero THD${}_{U}$ value; for a sinusoidal unbalanced source voltage, the THD${}_{U}$ value was approximately 10%; and for a non-sinusoidal balanced source voltage the THD${}_{U}$ value was approximately 6%. Neutral current was eliminated in all three cases.

### 2.4. Unity Power Vector

This method aims to consider the load in combination with the active power filter as a linear load along with the source. If this requirement is fulfilled, the source current after compensation can be described as

(7) $${i_{S}}_{ref}=k\times v_{s}=k\times V_{m}\sin\omega t,$$

where *k* is the conductivity of the nonlinear load and the power active filter. After compensation, the source current is sinusoidal, has the same shape as the source voltage, and is in phase. In addition, higher harmonics are suppressed, and the power factor is equal to one [16,68,69].

The reference current can be described as follows:

(8) $$\left[\begin{array}{c} {i_{S0}}_{ref}\\ {i_{S\alpha }}_{ref}\\ {i_{S\beta }}_{ref} \end{array}\right]=k\times \left[\begin{array}{c} v_{0}\\ v_{\alpha }\\ v_{\beta } \end{array}\right]=\frac{{\vec{p}}_{L\alpha \beta }+{\vec{p}}_{L0}}{{\left(v_{0}^{2}+v_{\alpha }^{2}+v_{\beta }^{2}\right)}_{DC}}\times \left[\begin{array}{c} v_{0}\\ v_{\alpha }\\ v_{\beta } \end{array}\right].$$

In a study by Montero et al. [16], the UPF theory was tested and compared with the iPQ theory, Synchronous Reference Frame (SRF), and PHC methods. A total of five scenarios were compared, and the methods were tested under different operating conditions (ideal voltage source, distorted and asymmetrical voltage source, different types of load distortion, etcetera). In the evaluation of these results, the current waveforms were identical to those of the voltage, and therefore did not correspond to the limits in IEEE 519, or were asymmetrical if the voltage was asymmetrical in nature. The immediate reactive power consumed by the load was eliminated. In conclusion, the authors stated that, unlike other methods, the limit values specified in IEEE 1459 were not met.

### 2.5. Perfect Harmonic Cancellation

This method can be considered as a combination of the iPQ, SRF, and UPF methods. The source current is therefore in phase with the basic component of the source voltage at the Point of Common Coupling (PCC) [16,68,70].

**Figure 5:**
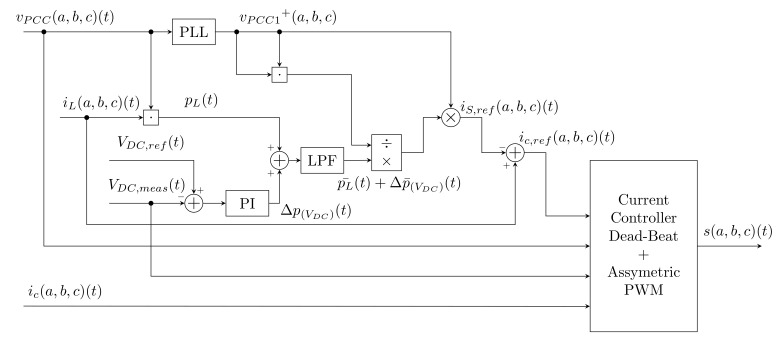
Diagram of Perfect Harmonic Cancellation.

For a block diagram of the Perfect Harmonic Cancellation method, see Figure 5.

The PHC method was tested by Montero et al. [16] in a study similar to the above-mentioned UPF method. Compared to the other tested methods, it provided the best results in all conditions (i.e., ideal or distorted conditions). The authors mentioned that this control method is designed to provide sinusoidal and symmetrical currents in phases with positive harmonic voltages. However, higher harmonic components or asymmetries nonetheless appear in PCC and the power factor does not achieve a value of 1. In conclusion, it they stated that if it is necessary to meet all standards regarding harmonics, asymmetry elimination, and reactive power compensation, PHC is the only control method (from iPQ, SRF and UPF) that meets the requirements for any conditions of use. This finding was verified experimentally by generating the worst-case scenario conditions (i.e., unbalanced distorted main voltage and unbalanced distorted current).

### 2.6. Synchronous Reference Frame

This method uses transformation from abc→dq coordinates, which causes the frames to rotate synchronously with the phase angle ϕ=ωt, where ω is the frequency of the source voltage. This is a widely used method [71,72,73,74,75,76,77,78,79,80,81,82,83,84]. This transformation is carried out by the following equation:

(9) $$\left[\begin{array}{c} i_{d}\\ i_{q} \end{array}\right]\;=\;\frac{2}{3}\left[\begin{array}{ccc} \sin\theta & \sin\left(\theta -\left(\frac{2\pi }{3}\right)\right) & \sin\left(\theta +\left(\frac{2\pi }{3}\right)\right)\\ \cos\theta & \cos\left(\theta -\left(\frac{2\pi }{3}\right)\right) & \sin\left(\theta +\left(\frac{2\pi }{3}\right)\right) \end{array}\right]\left[\begin{array}{c} i_{a}\\ i_{b}\\ i_{c} \end{array}\right],\;\begin{array}{l} \theta =\omega t. \end{array}$$

The components *d* and *q* represent the active and reactive components of the current. These currents can be therefore divided into

(10) $$\begin{array}{cc} i_{d} & =\bar{i_{d}}+\tilde{i_{d}},\\ i_{q} & =\bar{i_{q}}+\tilde{i_{q}}, \end{array}$$

where $\bar{i_{d}}$ and $\bar{i_{q}}$ are the fundamental harmonic components of the active and reactive components and $\tilde{i_{d}}$ and $\tilde{i_{q}}$ are the active and reactive components of the higher harmonic components.

**Figure 6:**
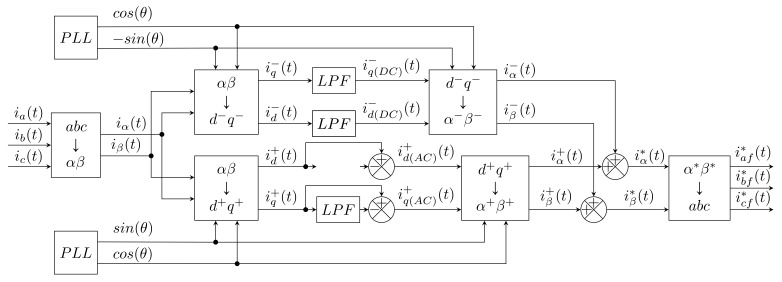
Block diagram of Synchronous Reference Method.

A low-pass filter is used to extract the fundamental harmonic component. In the *d-q* frames, the fundamental component appears as the *DC* component of the signal, while the higher harmonic components appear here as ripples or peaks. Subsequently, a reverse transformation is performed, and the result is waveforms of compensation currents (see Figure 6).

As with iPQ theory, this method is very sensitive to the quality of the source voltage, and it is necessary to measure three currents and the actual phase value.

**Figure 7:**
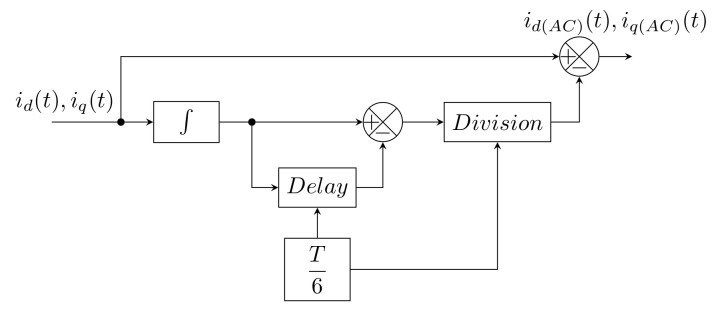
Modified SRF with eliminated LPF filter.

In [85], Oliveira introduces a modified SRF method. Here, it is assumed that even harmonic components are not part of the load current. Filters are replaced by blocks that calculate the floating average. In the *d-q* coordinates, the odd harmonic components are manifested as multiples of the number 6. More in [85]. Block diagram of this method can be seen in Figure 7.

The advantage over the classic SRF method is the absence of filters, subsequently generating delays. The delay takes a maximum of $\frac{1}{6}T$, where *T* is the period of the source signal. The method is immune to harmonic distortion in the supply voltage, and does not need to be adjusted in any way.

This method was not used in real implementation. In conclusion, it was only verified that the mathematical apparatus used in the modification of the SRF method is valid and functional. The method was designed primarily for a single-phase application, although it can of course be extended to a three-phase implementation.

### 2.7. Synchronous Detection Method

This method can successfully work in both balanced and unbalanced systems, as the compensation currents are calculated according to the voltage of individual phases. It can used to calculate compensation currents while a three-phase source supplies a highly nonlinear load [13,86,87,88,89].

The calculation of three-phase compensation currents using the method of uniform current distribution with the SDM algorithm is based on the assumptions that the voltage is not distorted and the loss in the neutral wire can be considered as negligible [13,86,87,88,89].

Assuming that the maximum values of the source currents are symmetrical after compensation,

(11) $$I_{am}=I_{bm}=I_{cm}=I_{m}.$$

Then, the maximum values of the active currents in each phase after compensation are

(12) $$\begin{array}{c} I_{am}=\frac{2P_{a}}{V_{am}},\;I_{bm}=\frac{2P_{b}}{V_{bm}},\;I_{cm}=\frac{2P_{c}}{V_{cm}}, \end{array}$$

where $P_{a}$, $P_{b}$, and $P_{c}$ are the active powers of each phase and $V_{am}$, $V_{bm}$, and $V_{cm}$ are the maximum values of the phase voltage of each phase.

The total average power is

(13) $$P_{av}=P_{a}+P_{b}+P_{c}.$$

By editing,

(14) $$P_{a}=\frac{V_{am}}{V_{t}}P_{av}\;,\;P_{b}=\frac{V_{bm}}{V_{t}}P_{av}\;,P_{c}=\frac{V_{cm}}{V_{t}}P_{av}.$$

The currents of the reference active source are then calculated as follows:

(15) $$\begin{array}{c} i_{ac\;ref}\left(t\right)=\frac{2p_{av}}{V_{am}V_{t}}v_{an}\left(t\right)\;,\\ i_{bc\;ref}\left(t\right)=\frac{2p_{av}}{V_{bm}V_{t}}v_{bn}\left(t\right)\;,\\ i_{cc\;ref}\left(t\right)=\frac{2p_{av}}{V_{cm}V_{t}}v_{cn}\left(t\right), \end{array}$$

where

(16) $$V_{t}=V_{am}+V_{bm}+V_{cm},$$

and the compensation currents are provided by

(17) $$\begin{array}{c} i_{ac}\left(t\right)=i_{an}\left(t\right)-i_{acc}\left(t\right)\;,\\ i_{bc}\left(t\right)=i_{bn}\left(t\right)-i_{bcc}\left(t\right)\;,\\ i_{cc}\left(t\right)=i_{cn}\left(t\right)-i_{ccc}\left(t\right). \end{array}$$

The *SDM* method was investigated in study by Sujatha et al. [90]. The method was tested in a simulation environment and the ability of the method to filter out higher harmonics in a balanced system as well as the response to dynamic changes in load power were investigated. In both cases, the THD${}_{I}$ improved sharply to 2.7%.

In addition, this method of control was discussed by Kabir et al. [86], who compared *SDM* with iPQ theory. In conclusion, they stated that compared to the iPQ theory, the *SDM* method is very slow. The iPQ theory takes less than one period to compensate, while the *SDM* method takes fourteen periods to compensate (280 ms at 50 Hz mains voltage). The comparison was performed only using simulations in the SIMULINK environment, and no practical comparison experiment was performed.

### 2.8. Cross-Correlation Technique

In this method, the fundamental harmonic of the current for each phase is calculated using correlation and cross-correlation coefficients. The calculation of $v_{s}$ and $v_{L}$ for each phase is as follows [91,92]:

(18) $$\begin{array}{c} {\left|\left|v_{s}\left(t\right)\right|\right|}^{2}=\frac{1}{T_{s}}\int_{t-T_{s}}^{t}{v_{s}}^{2}\left(t\right)\;dt\\ {\left|\left|i_{L}\left(t\right)\right|\right|}^{2}=\frac{1}{T_{s}}\int_{t-T_{s}}^{t}{i_{s}}^{2}\left(t\right)\;dt, \end{array}$$

where $T_{s}$ is the time duration of one cycle of $v_{s}\left(t\right)$. The product of $v_{s}$ and $i_{L}$ provides the following equation:

(19) $$\langle v_{s}\left(t\right),i_{L}\left(t\right)\rangle =\frac{1}{T_{s}}\int_{t-T_{s}}^{t}v_{s}\left(t\right)i_{L}\left(t\right)dt.$$

Finally, the fundamental harmonic current is calculated from the following equation:

(20) $$i_{Lp}=\frac{\langle v_{s}\left(t\right),i_{L}\left(t\right)\rangle }{{\left|\left|v_{s}\left(t\right)\right|\right|}^{2}}v_{s}\left(t\right).$$

**Figure 8:**
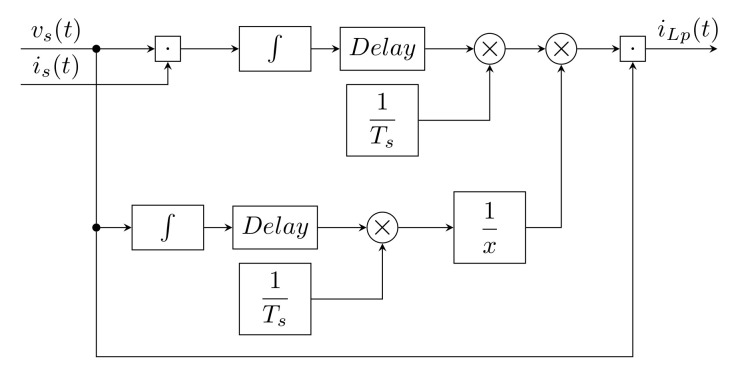
Block diagram of Cross-Correlation Technique.

Figure 8 shows a block diagram of a cross-correlation technique corresponding to the above equations.

Dunge et al. [92] examined the cross-correlation technique only in a simulation environment. The method was compared with modified SRF. Their comparison was performed on a model of a three-phase system with unbalanced load formed by a rectifier with an RL load. The results of their simulations showed the CCT method to have slightly better results than the SRF method, and the resulting THD${}_{I}$ was improved by about 20%. The results were evaluated in simulations based on *FFT* analysis and THD${}_{I}$ calculation.

### 2.9. Sine-Multiplication Theorem

This method is based on multiplication of a non-linear current with a reference sine current and subsequent integration. This method can be used in both single-phase and three-phase systems [20,93,94]. In the case of a three-phase system, three independent single-phase systems are controlled. The nonlinear load current is defined as

(21) $$i_{L}\left(t\right)=\sum_{n=1}^{\infty }I_{n}\sin\left(n\omega t+\theta_{n}\right).$$

This can be divided into a fundamental harmonic and harmonics as follows:

(22) $$i_{L}\left(t\right)=I_{1}\sin\left(\omega t+\theta_{1}\right)+\sum_{n=2}^{\infty }I_{n}\sin\left(n\omega t+\theta_{n}\right).$$

The reference sine signal is assumed as

(23) $$i_{r}\left(t\right)=\sin\left(\omega t\right).$$

The amplitude of the fundamental harmonic is obtained using the Fourier transformation, which corresponds to the following equation:

(24) $$I_{x}=\frac{1}{T}\int_{0}^{T}i_{L}\left(t\right)i_{r}\left(t\right)dt=I_{1}\cos\left(\theta_{1}\right).$$

Subsequently, the amplitude of the fundamental harmonic is calculated by multiplying $I_{x}$ and $i_{r}\left(t\right)$:

(25) $$i_{sc}\left(t\right)=I_{x}i_{r}\left(t\right)=I_{1}\cos\left(\theta_{1}\right)\sin\left(\omega t\right).$$

Finally, the compensation current is calculated by the following equation:

(26) $$\begin{array}{c} i_{cr}\left(t\right)=i_{L}\left(t\right)-i_{sc}\left(t\right)\;\\ =\sum_{n=1}^{\infty }I_{n}\sin\left(n\omega t+\theta_{n}\right)-I_{1}\cos\left(\theta_{1}\right)\sin\left(\omega t\right). \end{array}$$

**Figure 9:**
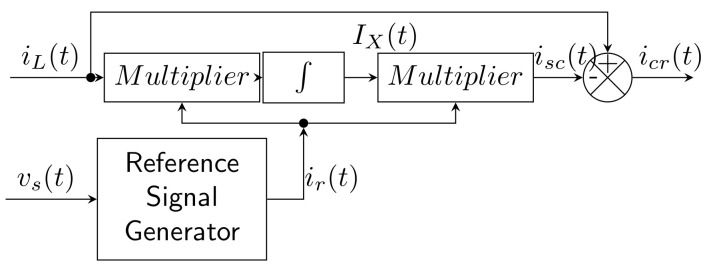
Block diagram of Sine-Multiplication Theorem.

Figure 9 shows a block diagram of Sine-Multiplication Theorem, where $V_{S}$ is the supply voltage.

In Jou et al. [93] the SMT was tested in simulation mode. The distorted current was successfully compensated even with a distorted source voltage (in this case, THD${}_{I}$ 12%). In conclusion, it was mentioned that the method is able to compensate for the power factor and suppress the harmonic components of symmetrical or asymmetrical loads.

### 2.10. Vectorial Formulation

Vector formulation does not require any transformations into other coordinate systems. An imaginary power component is calculated using power variables according to the following equation [14,17]:

(27) $$\vec{q}\left(t\right)=\vec{v_{q}}\times \vec{i}=\frac{1}{\sqrt{3}}\left[\begin{array}{c} v_{2}-v_{3}\\ v_{3}-v_{1}\\ v_{1}-v_{2} \end{array}\right]\left\lceil \begin{array}{c} i_{1}\\ i_{2}\\ i_{3} \end{array}\right\rceil \equiv \vec{q_{\alpha \beta }}\left(t\right).$$

The equation determining the currents are then calculated as follows:

(28) $$\left\lceil \begin{array}{c} i_{1}\\ i_{2}\\ i_{3} \end{array}\right\rceil =\frac{p}{u^{2}}\left\lceil \begin{array}{c} v_{1}\\ v_{2}\\ v_{3} \end{array}\right\rceil +\frac{1}{\sqrt{3}}\frac{p_{0}}{v_{0}^{2}}\left\lceil \begin{array}{c} v_{0}\\ v_{0}\\ v_{0} \end{array}\right\rceil +\frac{1}{\sqrt{3}}\frac{q}{u^{2}}\left[\begin{array}{c} v_{2}-v_{3}\\ v_{3}-v_{1}\\ v_{1}-v_{2} \end{array}\right].$$

Revuelta et al. [14,17,19,95] described various methods which were simulated and applied in three cases: sinusoidal balanced, sinusoidal unbalanced, and non-sinusoidal balanced source voltage. Applying the Vectorial Formulation achieved zero THD${}_{I}$ value in all three cases, and neutral current was eliminated in all three cases as well.

### 2.11. Conservative Power Theory

This theory is based on the definition of instantaneous power under non-sinusoidal conditions. It can be applied to single-phase and three-phase systems with or without a neutral wire. The advantage of this theory is that it does not require any transformation and all calculations are performed in the abc axis coordinate system [22,96,97,98,99]. The instantaneous active power component p(t) is provided by

(29) $$p\left(t\right)=v\times i.$$

The instantaneous reactive power component q(t) is provided by

(30) $$q\left(t\right)=\hat{v}\times i,$$

where $\hat{v}$ is the RMS value of the unbiased voltage integral. The average value of p(t) is the active component of the power:

(31) $$P=\langle v,i\rangle =\frac{1}{T}\int_{0}^{T}v\times i\;dT.$$

The average value of q(t) is the reactive component of the power:

(32) $$Q=\langle \hat{v},i\rangle =\frac{1}{T}\int_{0}^{T}\hat{v}\times i\;dT.$$

Based on the above equations, the currents are divided into the following three groups for each phase, namely, active phase currents:

(33) $$i_{a}=\frac{\langle v,i\rangle }{{\left|\left|v\right|\right|}^{2}}v,$$

reactive phase currents:

(34) $$i_{r}=\frac{\langle \hat{v},i\rangle }{{\left|\left|\hat{v}\right|\right|}^{2}}\hat{v},$$

and neutral line currents:

(35) $$i_{v}=i-i_{a}-i_{r}.$$

Calculation of currents in a balanced system is as follows:

(36) $$\begin{array}{c} i_{a}^{b}=\frac{\langle v,i\rangle }{{\left|\left|v\right|\right|}^{2}}v,\;\\ i_{r}^{b}=\frac{\langle \hat{v},i\rangle }{{\left|\left|\hat{v}\right|\right|}^{2}}\hat{v}. \end{array}$$

Calculation of currents in an unbalanced system is as follows:

(37) $$\begin{array}{c} i_{a}^{v}=i_{a}-i_{a}^{b},\;\\ i_{r}^{v}=i_{r}-i_{r}^{b}. \end{array}$$

Mortezaei et al. [22] performed a comparative study of three methods (iPQ, CPT, and SRF). The comparison was based on the results of simulations of several different electrical network distortion scenarios. Their results and conclusions showed that the CPT method is the most difficult of the three mentioned methods, has a better dynamic response (100 ms) than the iPQ method (140 ms), and is slow when compared to SRF method (50 ms).

Other publications that mention the use or comparison of the CPT method include [97,98,100,101,102].

### 2.12. Fourier Transform

Fourier transform is one of the most well known harmonic current extraction methods among the frequency-domain methods. According to [103], this method provides high accuracy in detection of harmonics and can be used in single-phase and three-phase systems. One of its advantages is that it only requires the use of current sensors. Disadvantages include the necessity of knowing at least one whole period of the current signal to estimate the reference current signal. The accuracy of this method is very sensitive to noise pollution of the signal and to the content of incomplete periods (*transient*) in the measured window. If the signal is symmetrical over the entire period, a half or quarter period can be used for calculations, however, these conditions are hard to achieve. Therefore, this method suffers from high computational burden and is very sensible to frequency changes [88,104,105,106,107].

Discrete Fourier Transform (DFT) is a mathematical transformation for discrete signals which returns information about both the amplitude and phase of the selected harmonic. When the higher harmonics in the current are detected and isolated, it is simply a matter of inverse transformation back to time domain. As mentioned, the method is very computationally demanding. This issue led to the development of Fast Fourier Transformation (FFT), which dramatically lowers the number of calculations from $N^{2}$ to $N\times \log_{2}\left(N\right)$, hence reducing the required DSP calculation time. The algorithm benefits from a decimation operation (in the time or frequency domain) that relies on recursive decomposition of an N-point DFT into two DFT transforms of N/2 points. Decomposition is repeated until the trivial “single-point” transform is reached and calculated [106]. When the frequency spectrum has been determined, the injection current is equal to summation of all sinus functions (with known amplitude, frequency, and phase).

Asiminoael et al. [106] carried out a comparison of three methods based on Fourier Transform, DFT, FFT, and the recursive variation of DFT. Their comparison was performed in a simulation environment, and the above-mentioned statements about DFT and FFT were confirmed. In addition, a comparison was made with iPQ, SRF, and generalized integrators. The authors stated that the main benefit of Fourier-based methods is avoiding the need to measure the voltage (reducing the required number of sensors) and the need to implement a numerical filter. Furthermore, FT methods perform very well under distorted voltage conditions. On the other hand, the calculation burden of FT methods is very high.

### 2.13. Recursive DFT

Recursive Discrete Fourier Transform (RDFT) [21,51] works on similar principles as DFT while recalculating the signal spectrum with each new sample. The Fourier coefficients are estimated in a window of samples with size N which is then shifted by the number of samples in *p*, where $p\in \left(1,N\right)$. As such, the estimate is calculated only from new samples in *p*. Therefore, RDFT is much less computationally demanding than DFT or FFT, and is much more suitable for real-time applications. The disadvantage of this method, as with DFT and FFT, is its sensitivity to noise and transitions between signal shapes. The accuracy and precision of this method depend on a sliding window.

**Figure 10:**
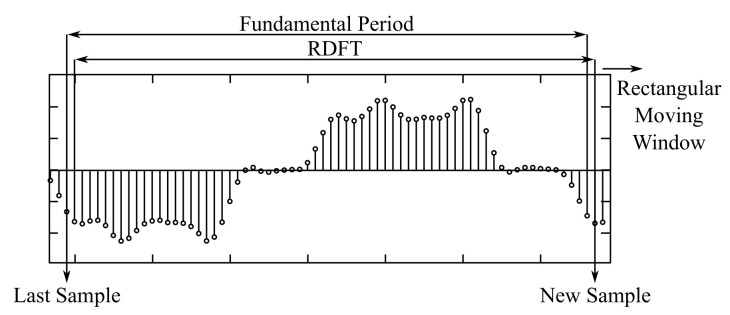
Recursive DFT principle.

In [21], the authors discussed the main property of RDFT, namely, that RDFT manages composite waveforms without an elaborate signal model in the frequency domain thanks to the implementation of floating-point correlation. Derivation of common industrial periodic waveforms is possible without knowledge of the physical model and its inputs. They further mentioned that with a suitable modification of the RDFT filter output (introduction of the window function), leakage of the spectrum can be reduced. In the end, the computational demands of RDFT were said to be very modest. Verification of these findings was performed in a simulation. The block diagram of this method can be seen in Figure 10.

### 2.14. Wavelet Transform

Wavelet Transform can be used in combination with the SRF method by replacing the LPF FIR filter by the wavelet transform to divide the signal into detailed components by a High-Pass Filter (HPF) and the approximation components by the Low-Pass Filter (LPF) [108]. The dominant approximation component is the fundamental harmonic component, and the detailed components contain harmonic components. This method is used mainly due to the fact that, unlike the FIR filter, there is no delay.

One publication where this method was used [109] compared it with the conventional window method using the window function. Furthermore, the methodology for suitably selecting the mother wavelet in terms of efficiency for transients, low overshoots, and oscillations in the frequency domain was discussed. Experiments were performed only by simulation, and the method was not verified on a real SAPF. The advantage of this method over the conventional SRF method is that the response of the filter created by WT is an order of magnitude faster compared to the conventional FIR filter and does not suffer from any overshoot. Applying the Hamming window function can further improve the results with WT. Db8 (Daubechies 8) was chosen as the parent wave in the above-mentioned study; the authors noted that the main disadvantage of this method is the need to know and use only one whole measuring window $T_{W}$.

**Figure 11:**
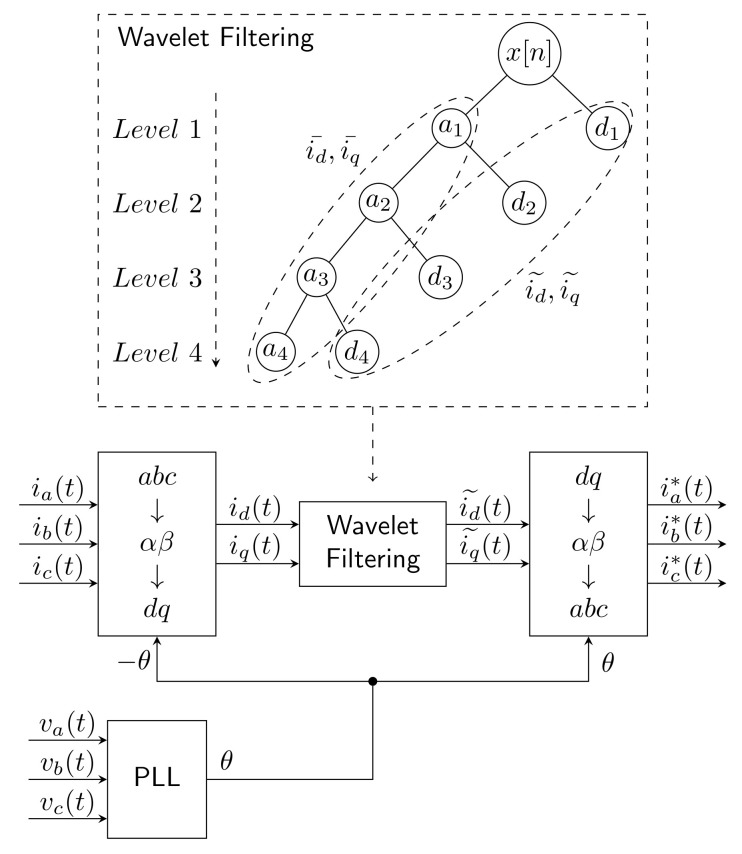
Wavelet Transformation principle.

The principle of this method is shown in Figure 11.

Driesen et al. [110] stated that the method is easy to implement and combines many advantages from several conventional methods. Advantages include the fact that the method can easily be used for single-phase systems and that it is applicable even under voltage-distorted conditions. In terms of dynamics, it has a very fast response to transients. However, neither experiments nor simulations were performed to verify the findings.

### 2.15. Hilbert Transform

The Hilbert Transform (HT) can be used to calculate the instantaneous properties of a signal. It is often used in combination with empirical mode decomposition as the Hilbert–Huang transform (HHT). It is most appropriate to apply HT to the signal after it has been processed, i.e., after noise filtering. The mathematical definition of HT is [111,112]:

(38) $$y\left(t\right)=\frac{P}{\pi }\int^{t}0\frac{x(\tau )}{t-\tau }d\tau ,$$

(39) z(t)=x(t)+jy(t),

where *P* is Cauchy principal value, z(t) is the analytic signal formed from the initial signal x(t) and its HT, the parameter a(t) is the instantaneous amplitude, and ϕ is the instantaneous phase [111,112].

Hilbert transform can be simplified as a convolution between the signal and $\left(\frac{1}{\pi }\right)t$. It can be implemented by an ideal filter with an amplitude response of unity and a phase response that is a constant ninety-degree delay. The Hilbert transform can be called a quadrature filter, as it shifts the phase of the spectral components by $\left(\frac{\pi }{2}\right)t$ [111,112].

The advantage of HT is that it is suitable for calculating the instantaneous frequency. The instantaneous frequency is an expression of the change in the instantaneous phase shift, and can be calculated using the analytical method. It is mathematically defined as follows [111,112]:

(40) $$\phi =\frac{1}{2\pi }\times \frac{d}{dt}\times \phi \left(t\right).$$

Aghazadeh et al. in 2013 [111] used HT to create a virtual phase and to transform a single-phase system into a two-phase symmetrical system. In their study, they presented a new method for harmonics elimination using a single-phase active filter. For evaluation, they used THD. Simulations were performed and the results showed that the new method in a single-phase AC railway system was more effective than the other compared methods.

In 2021, Swarnkar et al. [112] used HT in combination with Stockwell Transform (ST) to evaluate power quality (PQ) disturbances in a distribution system with availability of high-penetration of wind power generation. Their hybrid algorithm was designed to identify PQ issues using IPI and IPL notices. According to the results, the presented solution maintained better performance than a DWT-based method. Hilbert transform was used to process voltage signals during the evaluation process.

### 2.16. Kalman Filter

The Kalman Filter (KF) is a prediction-estimation algorithm that estimates the useful component of the signal according to previous values. This estimated signal is compared with the real signal, and the difference serves to improve the estimation of future samples. The basic Kalman filter can be described using two equations in the form of models [113,114,115].

Process model:

(41) $$x\left(n\right)=A\times x\left(n-1\right)+w\left(n\right),$$

where x(n) is a time-varying state variable, *A* is a state transition matrix, and w(n) is a process noise vector.

Measurement model:

(42) $$z\left(n\right)=H\times x\left(n\right)+v\left(n\right),$$

where z(n) is a measured signal, *H* is an observation matrix, and v(n) is the observation noise vector.

**Figure 12:**
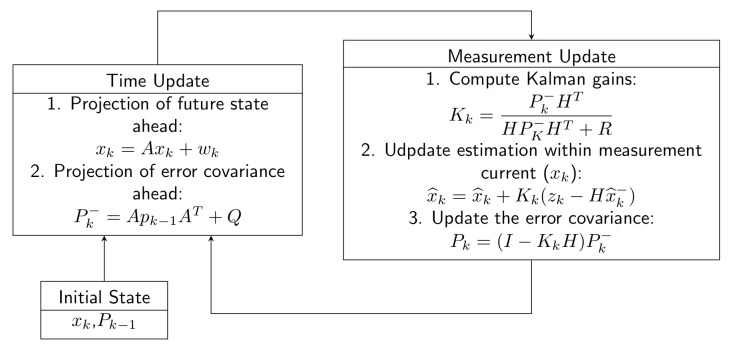
Structure of recursive calculation by prediction–correction method.

KF is performed in two phases, prediction and correction. The prediction phase is calculated according to previous data, and correction is then carried out according to a new measurement. Figure 12 shows a simple structural diagram of KF.

KFs may appear in different places in the SAPF structure. The first approach is based on the SRF method, in which a low-pass (LP) filter is used for extraction. The LP filter can be replaced by a KF to increase the robustness of the system. This is a suitable location, as the LP filter is a linear system. This strategy is used in [115]. The method was tested in both a simulated environment and a real experiment with RC load, and a comparison was made with a conventional low pass filter. The resulting THD${}_{I}$ was 0.42%, better than the low pass filter (with a value 2.38%), and met the THD${}_{I}$ limit values set by the IEEE 519 standard.

Another approach was described in [114]. This approach uses KFs to estimators the reference current and voltage. The current estimator with KF is based on the first ten odd current harmonics. The output of the estimator is the instantaneous value of the phase current; filtering out the fundamental harmonics produces the reference current. The voltage estimator with KF is based on the first five odd voltage harmonics. Together with DC-link voltage control, this provides a reference current which corresponds to the losses in the active filter. Combining the two reference currents produces the desired reference current. This method was tested by simulation in MATLAB, and it was found that THD${}_{I}$ decreased from 95.6% to 12.9%.

In other studies [113,116], KF has been used for reference current estimation $i_{a,b,c}^{*}$. This solution achieves better values compared to iPQ. In [113], the system was complemented by robust control (H-infinity). This connection leads to robustness of the whole system, simplification of the control structure, and reduction of the number of sensors (as there is no need to regulate the DC-link voltage using a separate PI controller). In 2018, Prince et al. [116] used a voltage regulator for DC-link and KF for current extraction. According to the results, the THD${}_{I}$ after compensation was 2.18%, compared to 30.56% before compensation. In 2017, Panigrahi [113] achieved a THD${}_{I}$ of 2.2% after compensation, compared to 25.9% before compensation.

### 2.17. Extended Kalman Filter

Extended KF (EKF) is usually used for nonlinear systems. The nonlinear system function is approximated using linear functions at the point of estimation (Taylor’s development). The basic system can be described by the process model provided in Equation (43) and the measurement model by Equation (44) [117,118,119,120]:

(43) $$x\left(n\right)=f\left(x\left(n-1\right),u\left(n-1\right),w\left(n-1\right)\right),$$

where x(n) is a time-varying state variable, *f* is the nonlinear system function, u(n) is an input, and w(n) is the process noise

(44) $$z\left(n\right)=h\left(x\left(n\right),v\left(n\right)\right),$$

where z(n) is the measured signal, *h* is the nonlinear function, and v(n) is the measurement noise.

**Figure 13:**
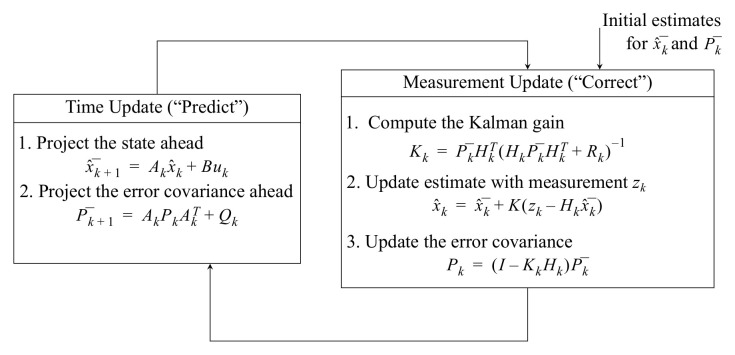
Simplified diagram of EKF algorithm for parameter estimation.

Similar to the basic KF, an EKF is divided into two phases. A simplified diagram is shown in Figure 13.

In [118], basic KF, EKF, and two other KF modifications (extended complex KF and robust ECKF) were described and compared. In all, cases THD${}_{I}$ was reduced from 24.9% to a value below 5%. There were no significant differences in THD${}_{I}$ achieved by the different KF types (THD${}_{I}=4.79$% was achieved by KF and THD${}_{I}=4.92$% was achieved by EKF). These results show the ordinary linear KF performing better than the EKF, while the RECKF algorithm achieved the best results (THD${}_{I}$ 4.46%).

### 2.18. Unscented Kalman Filte

There are other variants of KF, for example, the Unscented Kalman filter (UKF). Its strategy is not a linearization; rather, it uses direct nonlinearity. Currently, its deployment is not common in the SAPF framework. In one study [121], UKF was used to estimate individual power and frequency components. UKF showed improved the performance, especially with respect to transient phenomena in the network.

### 2.19. Adaptive Linear Neuron

**Figure 14:**
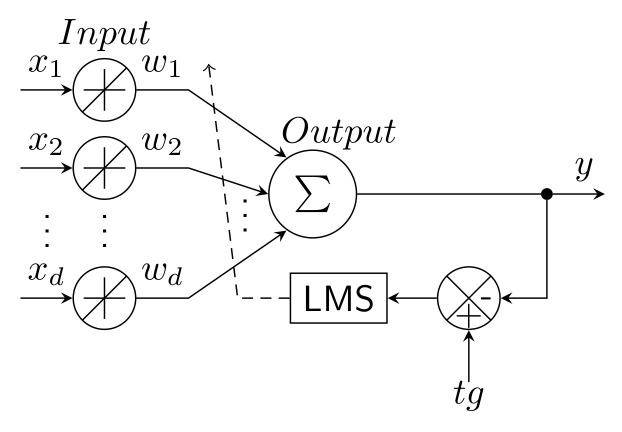
ADALINE schematic.

ADALINE is an abbreviation for Adaptive Linear Neuron, a neural network that has two layers, input and output. It can estimate functions that have linear dependence between its input and output, and retains its functionality in certain nonlinear real applications [122]. Inputs $x_{i}$ are multiplied by adjustable weights $w_{i}$, then a sum is derived from these multiples. The estimated output *y* is compared with a reference value tg (target) and the resulting error *e* is used by the Least Mean Squares (LMS) algorithm to train the network weights. A design scheme of the algorithm is shown in Figure 14.

It is assumed that the current flowing through the load $i_{L}\left(t\right)$ is a distorted signal [23], where $f_{1}$ is a fundamental harmonic frequency of the grid and $a_{k}$ and $b_{k}$ are the harmonic sine and cosine amplitudes, respectively. Assuming that the value of $f_{1}$ must be known, the sine and cosine components can be easily calculated. However, the amplitude values of $a_{k}$ and $b_{k}$ are unknown variables and must be estimated using the ADALINE algorithm. The sine and cosine components can be thought of as inputs to the ADALINE equation, with the individual amplitudes $a_{k}$ and $b_{k}$ as network weights. The output of the network is *y*, which in this case is the estimated current $i_{L}$ flowing through the load. The measured current flowing through the load serves as the reference value tg. The estimated current flowing through load *y* can be written as

(45) $$y^{n}=\sum_{k=1,5,7,11\ldots }^{H}a_{k}^{n}\sin\left(2\pi kf_{1}t^{n}\right)+b_{k}^{n}\cos\left(2\pi kf_{1}t^{n}\right),$$

where $y^{n}$ is sampled by time $t_{n}=nT_{s}$ (where *n* is the sample index and $T_{s}$ is the sampling period), *k* is the harmonic component index, and *H* is the value of the highest examined harmonic component. Components that are even or multiples of three are omitted due to half-wave symmetry and three-phase connection [23].

Individual scales are trained by the LMS algorithm, which is based on the optimization of weights using Gradient Descent (GD). The GD process is applied to minimize the square of the error between the reference $tg^{n}=i_{L}^{n}$ and the estimated output $y^{n}$. The criterion can be then expressed as follows:

(46) $$E=\frac{1}{2}{\left(e^{n}\right)}^{2}=\frac{1}{2}{\left(i_{L}^{n}-y^{n}\right)}^{2}=\frac{1}{2}{\left(i_{L}^{n}-{\left(w^{n}\right)}^{T}x^{n}\right)}^{2}.$$

Using the gradient value ∇E with respect to the weight vector $w^{n}$, the resulting expression appears as follows:

(47) $$\nabla E\left(w^{n}\right)=-e^{n}\times x^{n}.$$

The rule for weight adjustment then appears as

(48) $$w^{n}=w^{n-1}-\eta \times \nabla E^{n}\left(w\right)=w^{n-1}+\eta \times e^{n}\times x^{n},$$

where η is the step size or learning rate of the weight update.

In 2011, Bhattacharya and Chakraborty [29] used ADALINE to estimate the compensation current in order to improve the convergence time and reduce the computational demands of the method. The THD${}_{I}$ criterion was used to evaluate the quality of filtration. The neural network weights were adjusted so that the THD${}_{I}$ reached its local minimum. Simulations in the SIMULINK environment were used in experiments performed under laboratory conditions. The results confirmed that the proposed solution was functional for both symmetrical and asymmetrical loads in a three-phase three-wire system. The experiment revealed that the use of two weights and two input vectors helped it to converge very quickly.

In 1996, Dash et al. [123] used ADALINE to estimate Fourier coefficients in a signal polluted with noise, higher harmonics, and DC components. The learning parameters of the neurons were set to minimize the error between the measured and desired current signals. The size of the weights was estimated using LMS and nonlinear weight settings. Using this algorithm, an adaptive search for higher harmonic components was easily achieved. Compared to the backpropagation approach, better management stability and faster convergence times were attained. The experiments were simulated in MATLAB and verified on a real platform. The ADALINE method was compared with other algorithms based on the Kalman filter and DFT, and showed significantly better quality. Thanks to its adaptability, it was stated that this method is suitable for finding higher harmonic components where both the amplitude and the phase angle often change.

In 2007, Abdeslam et al. [26] designed and tested a complex system where ADALINE was used in an SAPF application to solve three different problems, namely, extraction of voltage harmonic components to regain a symmetrical and balanced network, filtering of higher harmonic components, and control of the SAPF power circuit. Using a unified system of methods, higher efficiency was achieved and the quality of filtration was improved. The main advantage of this approach is the ability to adapt in real time to varying types of loads. The experiments were verified in both the SIMULINK simulation environment and in a real scenario. The authors highlighted the advantages arising from the use of one ADALINE method for various problems within one SAPF system.

In a 2014 study, Qasim et al. [23] compared the ADALINE method with the feed-forward Multilayer Neural Network (MNN) method, another frequently used method for SAPF control. ADALINE was trained during operation using the LMS algorithm, while the MNN was pre-trained using the Scale Conjugate Gradient (SCG) backpropagation algorithm. The task was to extract the amplitude of the fundamental harmonic component of the current flowing through the load. Both algorithms were verified in simulations as well as through experiments. Both the obtained results and the conclusion of the study showed the ADALINE method to be more suitable for SAPF management than MNN. This was especially true in scenarios where an unlearned load was controlled; the MNN method failed to extract the basic component, which resulted in overcompensation by the PI controller. In contrast, ADALINE can be used in any scenario, as it learns adaptively during its operation.

### 2.20. Adaptive Neuro-Fuzzy Inference System

**Figure 15:**
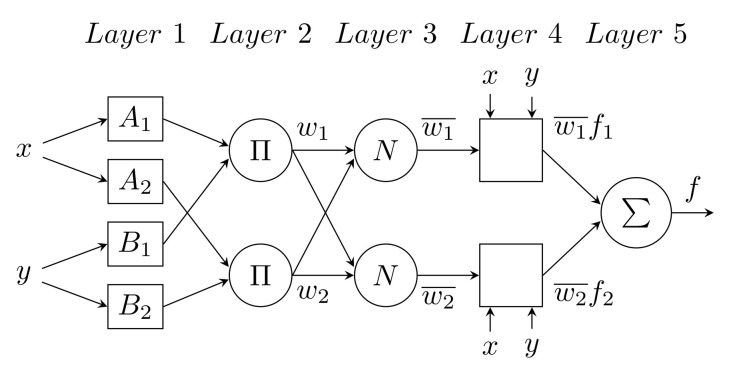
ANFIS architecture schematic with two rules.

The Adaptive Neuro-Fuzzy Inference System (ANFIS) is a method based on a fuzzy expert system implemented by a multilayer forward neural network. Functionally, it is equivalent to a fuzzy inference system of the Takagi–Sugeno [124] type. The two-input ANFIS rule basis contains two Fuzzy IF–THEN T-S rules of the following form:

(49) $$\mathrm{IF}\;x\;\mathrm{is}\;A_{i}\;\mathrm{and}\;y\;\mathrm{is}\;B_{i}\;\mathrm{THEN}\;f_{i}=p_{i}x+q_{i}y+r_{i}$$

where *x* and *y* are inputs, $A_{i}$ and $B_{i}$ are fuzzy sets, $f_{i}$ are outputs of the fuzzy system, and $p_{i}$, $q_{i}$, and $r_{i}$ are setting parameters that are obtained by estimation during the learning process. The ANFIS architecture for implementing these two rules is shown in Figure 15 [125]. The squares indicate a fixed node, while the circles indicate an adaptive one. ANFIS consists of a total of five layers; the function of the nodes in a given level is of the same type. A more detailed description of the ANFIS architecture shown in Figure 15 can be found in [126]).

The parameters for setting the FIS are of two types: non-linear in the first layer and linear in the fourth layer. First, it is necessary to initialize the Fuzzy Inference System (FIS) approximately, which is then set up by the ANFIS adaptive learning process. ANFIS uses the original FIS and sets it up using a hybrid learning technique combining the feedback gradient method and the least squares method. The result of each iteration of learning is an error that is gradually reduced. Learning ends when the set number of iterations is reached or the system error is within acceptable limits. The gradient method is usually used to set the input layer and the least squares method is used to optimize the parameters in the defuzzification layer.

In the learning algorithm, each iteration repeats a forwards and backwards run. In the forwards run, training patterns are fed to the input (*X* and *Y* in the general case), then the outputs of the individual layers are gradually calculated, and finally the resulting parameters are identified by least squares estimation. The output vector *Z* of a linear function has the shape

(50) $$Z_{i}=m_{i}X+n_{i}Y+q_{i}.$$

In 2013, Routh and Arun [24] compared three methods to improve power quality, suppress the reactance component of the power, and compensate for higher harmonics at nonlinear loads. The control was performed using a PI controller, a fuzzy controller, and ANFIS. The three methods were compared via simulations. The results showed ANFIS with the best results in terms of THD${}_{I}$, and it was able to compensate for even the largest deviations.

ANFIS was used in a 2013 study [127] to manage a three-phase SAPF as a cost-effective solution to power quality problems. The filter showed good results at both the steady state and under dynamically changing conditions. The advantages of the proposed control system were mainly the independence of the distortion and asymmetry of the input source voltage, fast and accurate extraction of the fundamental harmonic component under both symmetrical and asymmetrical conditions, and the ease and simplicity of implementing the architecture. The results showed that SAPF was able to reduce THD${}_{I}$ to tenths of a percent, which fully complies with the IEEE-519 standard. It was compared with a fuzzy controller and ANFIS, with the results showing improvement of up to 50% compared to the other controllers.

In 2013, Martinek et al. [128] used ANFIS to control the drive to meet SAPF functionality. ANFIS was successfully implemented; its functionality in terms of suppression of higher harmonic components was verified and a comparison of various algorithm settings. A total of four ANFIS models were presented, which differed in their number of nodes (21–75), number of linear parameters (12–75), number of nonlinear parameters (12–30), and number of fuzzy rules (4–25). The investigated phases of the network reached THD${}_{I}$ values of approximately 98%; the first model, Model A, suppressed THD${}_{I}$ to approximately 9%, while the remaining Models B, C, and D were able to suppress THD${}_{I}$ below approximately 1%, which fully complies with IEEE-519 and IEC-61000-3 standards. In conclusion, it was found that this method provides clearly better results than the iPQ method, is easier to implement on a microprocessor, and can cope with frequency changes due to network instability. System learning was performed offline. The authors further stated that the subject of their future research was to adjustment the system to allow it to be taught during active processes, and thus become able to cope with new network pollution conditions.

### 2.21. Least Mean Squares Algorithm

**Figure 16:**
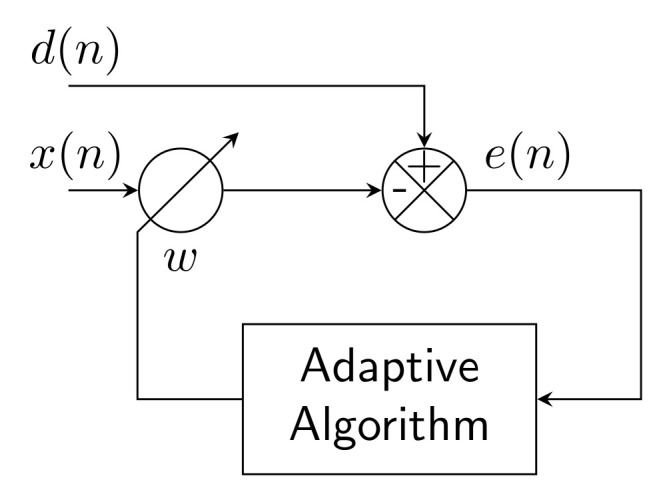
Schematic of an adaptive algorithm.

Figure 16 shows a block diagram of an adaptive filter for use in an unknown or time-variant environment which is unpredicted. For this structure, Finite Impulse Response (FIR) or Infinite Impulse Response (IIR) are used with filters of adaptive coefficients which vary in time. These filters, which are controlled by adaptive algorithms, are called adaptive filters. Various adaptive algorithms [129] from the LMS and RLS families have been used in this application, and are described below. The required inputs for adaptive filters are the reference signal x(n) and input signal d(n). The outputs from adaptive filters are the desired signal y(n) and error signal e(n), where *n* is the number of iterations.

The LMS algorithm is the main representative of a class of stochastic gradient algorithms based on the Wiener theory and the method of least squares. This method is widely used due to its simplicity, low computational complexity, and robustness. Each iteration of the LMS algorithm requires three different steps to be performed in a specified order [25,130,131].

The output of an FIR filter $y\left(n\right)$ is calculated as follows:

(51) $$y\left(n\right)={\vec{w}}^{T}\left(n\right)\times \vec{x}\left(n\right)=\sum_{i=0}^{N}w_{i}\left(n\right)\times x\left(n-i\right),$$

while the value of the estimated error $e\left(n\right)$ is calculated as

(52) $$e\left(n\right)=d\left(n\right)-y\left(n\right).$$

In the end, the weights of the filter vector $\vec{w}\left(n\right)$ are updated with respect to the following iteration:

(53) $$\vec{w}\left(n+1\right)=\vec{w}\left(n\right)+2\times µ\times e\left(n\right)\times \vec{x}\left(n\right).$$

The parameter µ is called the convergence constant or step size of the LMS algorithm. It is a small positive constant with a value generally less than 1, and affects the adaptive properties of the algorithm. Larger step size values result in a loss of stability and inaccurate filtration. Smaller values increase computation time and demands and extend the convergence time.

There are modifications to the LMS algorithm that are worth mentioning, such as Normalized LMS [132,133] and Sparse LMS [134]. The NLMS algorithm has a variable convergence constant for each iteration, which improves the convergence rate of the adaptive filter. The SLMS algorithm involves fewer multiplication operations than the LMS, although it has a slower convergence rate and higher steady state error.

In [132], our team used simulations to examine the ability of adaptive LMS and RLS algorithms to compensate for higher harmonic components of the current. We assumed 100µs as the theoretical delay of the inverter. After applying the LMS algorithm, the THD${}_{I}$ value was reduced from 63.27% to 12.51%. The step size µ had a great influence on the quality of compensation, making it necessary to find a compromise between the THD${}_{I}$ value and the convergence time.

### 2.22. Recursive Least Squares

The Recursive Least Squares (RLS) algorithm is the main representative in a class of recursive algorithms based on Kalman filtering theory and the least squares method. Unlike the LMS algorithm, it has its own statistical concept. The RLS algorithm works with average values calculated from time developments. The structure of the filter remains the same as with the LMS algorithm; only the adaptive process is different, due to the use of averages. Each iteration of the RLS algorithm requires five different steps to be performed in a specified order [25,130,131].

The output of the FIR filter y(n) is calculated as follows:

(54) $$y\left(n\right)={\vec{w}}^{T}\left(n\right)\times \vec{x}\left(n\right)=\sum_{i=0}^{N}w_{i}\left(n\right)\times x\left(n-i\right).$$

The medium-range vector gain $\vec{k}\left(n\right)$ is updated with respect to the following iteration:

(55) $$\vec{k}\left(n\right)=\frac{P\left(n-1\right)\times \vec{x}\left(n\right)}{\lambda +{\vec{x}}^{T}\left(n\right)\times P\left(n-1\right)\times x\left(n\right)}.$$

The value of the estimated error $e\left(n\right)$ is calculated as

(56) $$e\left(n\right)=d\left(n\right)-y\left(n\right).$$

The weights of the filter vector $\vec{w}\left(n\right)$ are updated with respect to the following iteration:

(57) $$\vec{w}\left(n\right)=\vec{w}\left(n-1\right)+e\left(n\right)\times k\left(n\right).$$

In the end, the inverse matrix P(n) is calculated as follows:

(58) $$P\left(n\right)=\lambda^{-1}\times P\left(n-1\right)-\lambda^{-1}\times k\left(n\right)\times {\vec{x}}^{T}\left(n\right)\times P\left(n-1\right).$$

The functionality of the RLS algorithm is fundamentally influenced by the choice of the forgetting factor λ, taking the values $\lambda \in \left(0,\right.\left.1\right]$. Assuming that λ=1, it is possible to use an estimate without forgetting. In practical implementation, λ from λ=0,98 to λ=1 is usually considered.

There is a modification of the RLS algorithm called QR-RLS (or QRD RLS). This modification uses a triangulation process and has good mathematical properties thanks to robust QR decomposition.

In [132], our team examined the ability of adaptive LMS and RLS algorithms in simulations to compensate for higher harmonic components of the current. We assumed 100 µs as the theoretical delay of the inverter. After applying the RLS algorithm, the THD${}_{I}$ value was reduced from 63.27% to 6.43%. The forgetting factor λ had a certain effect on the quality of the compensation, determining how many samples the algorithm can remember. For λ values in the range of 0.999 to 1, there was not a significant difference in THD${}_{I}$ or SNR values; however, at a λ value of 0.99 there was a high THD${}_{I}$ value, meaning that the system was unstable.

### 2.23. Notch LMS Algorithm

**Figure 17:**
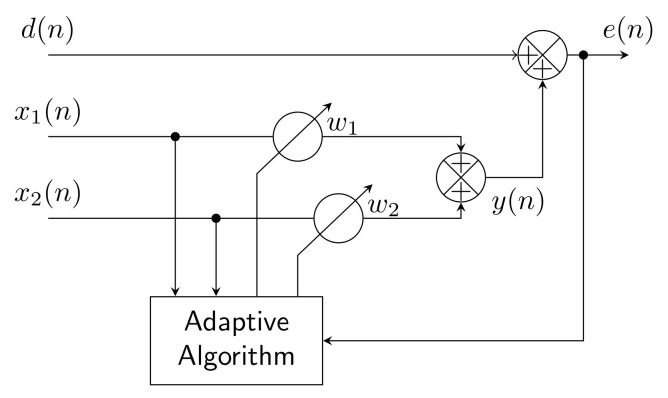
Block diagram of the notch adaptive filter.

Figure 17 shows a block diagram of a notch adaptive filter. The adaptive filter using this architecture is able to work with multiple reference input signals from difference sources. One transverse FIR filter weight for a vector coefficient $w_{i}$ is needed for each reference input signal [135,136].

The notch LMS is a modification of an LMS algorithm for a notch adaptive filter structure. This method is used in a three-phased system. As with the LMS algorithm, the notch LMS algorithm requires three different steps to be performed in a specified order [135,136]:

The output of the FIR filter $y\left(n\right)$ is calculated as follows:

(59) $$y\left(n\right)={\vec{w}}_{1}^{T}\left(n\right)\times {\vec{x}}_{1}\left(n\right)-{\vec{w}}_{2}^{T}\left(n\right)\times {\vec{x}}_{2}\left(n\right),$$

and the value of the estimated error $e\left(n\right)$ is calculated as

(60) $$e\left(n\right)=d\left(n\right)-y\left(n\right).$$

In the end, the weights of the filter vectors ${\vec{w}}_{1}\left(n\right)$ and ${\vec{w}}_{2}\left(n\right)$ are updated with respect to the following iteration:

(61) $$\begin{array}{c} {\vec{w}}_{1}\left(n+1\right)={\vec{w}}_{1}\left(n\right)+2\times µ\times e\left(n\right)\times {\vec{x}}_{1}\left(n\right),\\ {\vec{w}}_{2}\left(n+1\right)={\vec{w}}_{2}\left(n\right)+2\times µ\times e\left(n\right)\times {\vec{x}}_{2}\left(n\right). \end{array}$$

In a previous publication of our team [135], this method was implemented on an FPGA and its functionality was verified in practice through a real experiment. The THD${}_{I}$ value was reduced from values around 25% to values around 1% at low values of the parameter µ. The results of this experiment showed that the size of µ directly affects both the quality of the resulting filtration (THD${}_{I}$ value) and its dynamics. At higher values of µ, the adaptation time decreases (higher dynamics of the system), however, the suppression of harmonic components is not effective (up to 13% in the experiment).

### 2.24. Notch RLS Algorithm

The notch LMS is a modification of the RLS algorithm for a notch adaptive filter structure. This method is used in a three-phased system. As with the RLS algorithm, the notch RLS algorithm requires five different steps to be performed in a specified order [135,136]:

The output of the FIR filter $y\left(n\right)$ is calculated as follows:

(62) $$y\left(n\right)={\vec{w}}_{1}^{T}\left(n\right)\times {\vec{x}}_{1}\left(n\right)+{\vec{w}}_{2}^{T}\left(n\right)\times {\vec{x}}_{2}\left(n\right),$$

and the medium-range vector gains ${\vec{k}}_{1}\left(n\right)$ and ${\vec{k}}_{2}\left(n\right)$ are calculated as

(63) $$\begin{array}{c} {\vec{k}}_{1}\left(n\right)=\frac{P_{1}\left(n-1\right)\times {\vec{x}}_{1}\left(n\right)}{\lambda +x_{1}^{T}\left(n\right)\times P_{1}\left(n-1\right)\times {\vec{x}}_{1}\left(n\right)},\\ {\vec{k}}_{2}\left(n\right)=\frac{P_{2}\left(n-1\right)\times {\vec{x}}_{2}\left(n\right)}{\lambda +x_{2}^{T}\left(n\right)\times P_{1}\left(n-1\right)\times {\vec{x}}_{2}\left(n\right)}. \end{array}$$

The value of estimated error $e\left(n\right)$ is calculated as

(64) $$e\left(n\right)=d\left(n\right)-y\left(n\right).$$

The weights of the filter vectors ${\vec{w}}_{1}\left(n\right)$ and ${\vec{w}}_{2}\left(n\right)$ are updated with respect to the following iteration:

(65) $$\begin{array}{c} {\vec{w}}_{1}\left(n\right)={\vec{w}}_{1}\left(n-1\right)+e\left(n\right)\times k_{1}\left(n\right),\\ {\vec{w}}_{2}\left(n\right)={\vec{w}}_{2}\left(n-1\right)+e\left(n\right)\times k_{2}\left(n\right). \end{array}$$

In the end the inverse matrices $P_{1}\left(n\right)$ and $P_{2}\left(n\right)$ are calculated as follows:

(66) $$\begin{array}{c} P_{1}\left(n\right)=\lambda^{-1}\times P_{1}\left(n-1\right)-\lambda^{-1}\times \\ k_{1}\left(n\right)\times {\vec{x}}^{T}\left(n\right)\times P_{1}\left(n-1\right),\;\\ P_{2}\left(n\right)=\lambda^{-1}\times P_{2}\left(n-1\right)-\lambda^{-1}\times \\ k_{2}\left(n\right)\times {\vec{x}}^{T}\left(n\right)\times P_{2}\left(n-1\right). \end{array}$$

In [135], our team implemented notch RLS in combination with Clarke transformation in simulations. We simulated a 100 µs delay of the inverter and examined THD${}_{I}$ and SNR values in a scenario where the current changed in a specified order for 160 s. The relative improvement in THD${}_{I}$ was about 20% and the improvement in SNR was about 10%.

### 2.25. Conclusions of Harmonic Extraction Algorithms

The choice of a Harmonic Extraction Algorithm depends mainly on the relevant voltage properties. If the voltage is ideal, i.e., undistorted and balanced, then it is possible to use simple algorithms from the time domain, such as iPQ theory, Cross-Vector Theory, PQR Theory, and others, or FFT, DFT, and RDFT from the frequency domain. These algorithms are easy to implement and computationally undemanding. However, if the voltage is distorted and unbalanced, these methods have degraded functionality.

In real conditions, where voltage is distorted and unbalanced, it is necessary to use more complex algorithms that work properly even under non-ideal conditions. These complex algorithms are divided into time domain, frequency domain, and adaptive algorithms. Time domain algorithms mostly require voltage measurement, i.e., they require more measuring probes. Frequency domain algorithms require a whole period of measured current, which causes a compensation delay. Adaptive algorithms are the most computationally demanding of the three.

Another important feature of Harmonic Extraction Algorithms is the need to set method parameters. While time domain methods do not require set parameters, other methods require optimal settings to fulfill their required functionality. A major issue is that most of methods do not have a clear methodology for setting the parameters, meaning that parameters must be set using a heuristic approach, which can be time-consuming to perform. All methods are designed mainly for a three-phase network, although several of them can be used in a single-phase network as well. A summary of the methods mentioned above can be found in Table 1.

## 3. Synchronisation Techniques

This section describes methods for obtaining a synchronization waveform, which is then used in SAPF to generate a current compensation signal. In order for the functionality to be correct, it is necessary to ensure that the phase of the current compensation signal is identical to the phase of the fundamental component of the source voltage, meaning that both signals are in phase. Only then can the current signals (compensation and original) be summed correctly, and therefore correctly compensated. A total of seven methods are discussed in detail, most of which are based on PLL.

### 3.1. Zero-Cross Detection

Zero-Cross Detection (ZCD) is one of the earliest and simplest synchronization algorithms [42,45,48]. When the voltage intersects the 0V value, a pulse is generated. The biggest advantage of the algorithm is a very simple implementation, while a significant disadvantage is its sensitivity to any noise and distortion by higher harmonics [30]. In this case, many zero crossings can occur, which can lead to impaired detection. Filtering may be performed before ZCD to improve the results [38]. However, this can cause phase deflection (i.e., the signal is preceded or delayed), and because the detector operates in the analogue domain, it is difficult to deal with this problem. The detector is not fast in terms of dynamics, as the zero crossing occurs only twice per period of the signal under investigation [47]. The implementation of this algorithm (in analogue form) is expensive, as for each phase a separate device must be created to monitor the zero crossing. For example, in [42], a ZCD circuit is designed to generate an initialization pulse for the SAPF control circuit when a zero crossing is detected at the PCC. This technique can be used for both one-phase [42] and three-phase [45] systems. However, due to its disadvantages, it is the least used synchronization technique among the various alternatives for SAPF applications.

### 3.2. Space Vector

**Figure 18:**
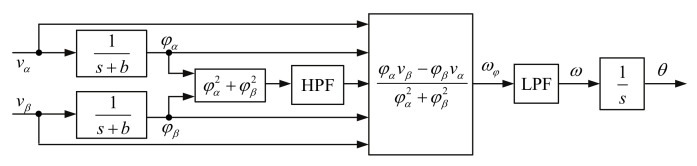
Space vector method.

Space vector (SV) is another synchronization method; it essentially detects the angular frequency of the space vector $v=v_{\alpha }+jv_{\beta }$. It obtains the instantaneous angular frequency by the equation below, where the space vector $\varphi =\varphi_{\alpha }+j\varphi_{\beta }$ is calculated using the transfer function $\varphi \left(s\right)=\frac{1}{s+b}v\left(s\right)$. The parameter *b* is the damping coefficient used for stability. The advantage of this method is that it is less sensitive and is robust to interference. On the other hand, the SV method is very sensitive to input frequency changes. The SV method has been used in several studies [137,138,139]. The block scheme of the SV method is shown in Figure 18.

(67) $$\omega_{\varphi }=\frac{\varphi_{\alpha }v_{\beta }-\varphi_{\beta }v_{\alpha }}{\varphi_{\alpha }^{2}v_{\beta }^{2}}$$

### 3.3. Phase-Locked Loop

This is the most well known and widely used synchronization technique thanks to its ease of control and ability to respond to changes in the distribution network. It is a relatively old method, and the concept was been considered as early as 1930 [140].

**Figure 19:**
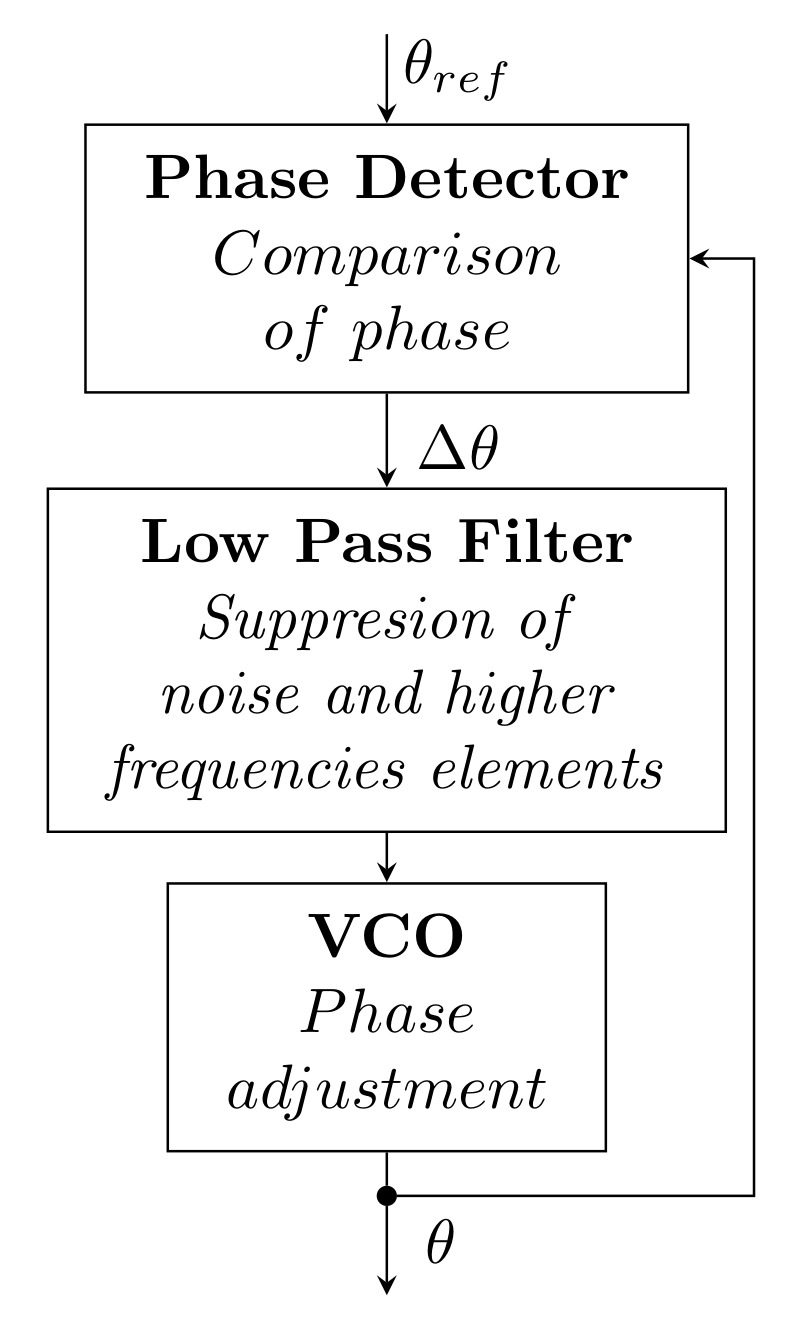
Function diagram of Phase-Locked Loop algorithm.

Its structure consists of three basic blocks (see Figure 19): a Phase Detector (PD), Loop Filter (LF), and Voltage-Controlled Oscillator (VCO). The PD first compares two input signals, namely, a reference signal with phase $\theta_{ref}$ and a feedback signal with phase θ, then generates the phase difference Δθ. The resulting error is then filtered using LPF, which removes noise and other higher frequency components from the PD. This signal is processed by the VCO, which generates a new value of the phase θ, which is then fed back to the PD. The process is repeated until the phase error is zero or close to zero. When this condition is met, the phase is locked and the generated phase θ corresponds to the setpoint $\theta_{ref}$.

#### 3.3.1. Synchronous Reference Frame PLL

**Figure 20:**
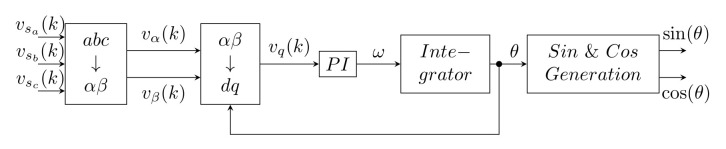
Three-phase SRF-PLL.

The conventional PLL described above can be further extended to SRF-PLL [34,40,44], which can be applied in both single-phase [28,31,40] and three-phase [28,34,37,44] applications. The main difference compared to the conventional PLL method is the implementation of the phase detection block, as seen in Figure 20.

**Figure 21:**
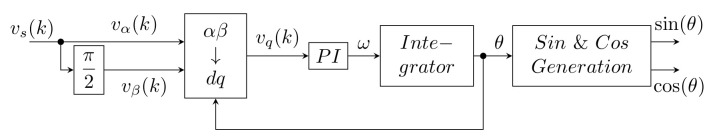
One-phase SRF-PLL.

For single-phase systems (Figure 21), the step with the Clark transformation is omitted and the β component is replaced by the α component, which is shifted by π/2. In this case, the α component refers specifically to the phase voltage signal.

This is an undemanding implementation method [37], exhibiting synchronization accurate results under ideal network conditions [34,37]. For proper operation it is necessary to observe the optimum settings for the PI controller and the least possible distortion of the network sinusoid. Improvement can be achieved by inserting the LPF after the transformation block [35]. However, it is necessary to set the filter order and cut-off frequency appropriately in order to achieve suitable distortion suppression and low delay.

To suppress these shortcomings, the method has been modified and extended by a self-tuning filter on STF-PLL [40] and by decoupled double SRF-PLL, i.e., DDSRF-PLL [44]. These adjustments allow the method to be used under conditions where the network is distorted or asymmetrical.

#### 3.3.2. Self-Tuning Filter PLL

**Figure 22:**
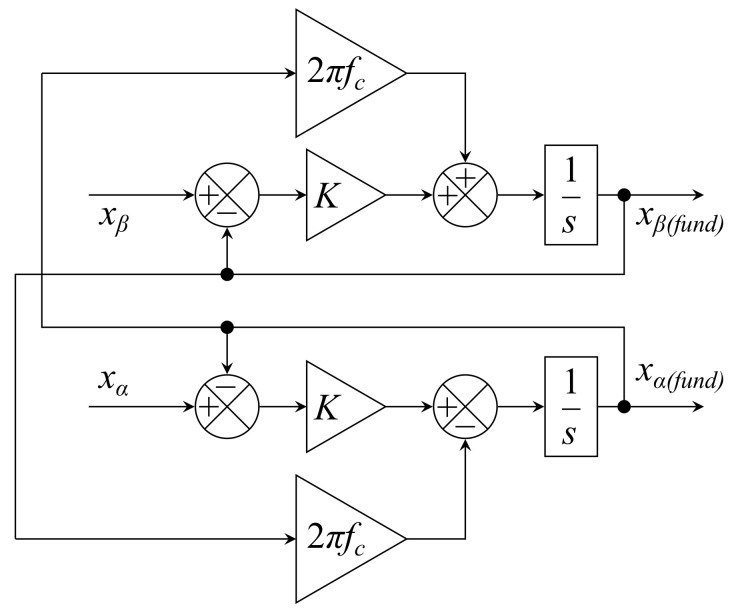
Self-tuning filter principle diagram.

Before transforming into dq, the αβ inputs are first filtered using STF, which suppresses noise and other high frequency components. The signal is therefore distortion-free and can be processed by the PLL without a detection error, as follows:

(68) $$\left[\begin{array}{c} x_{\alpha (fund)}\left(s\right)\\ x_{\beta (fund)}\left(s\right) \end{array}\right]\;=\;\frac{K}{s}\left[\begin{array}{c} x_{\alpha }\left(s\right)-x_{\alpha (fund)(s)}\\ x_{\beta }\left(s\right)-x_{\beta (fund)(s)} \end{array}\right]\;+\frac{2\pi f_{c}}{s}\left[\begin{array}{c} -x_{\beta (fund)}\left(s\right)\\ x_{\alpha (fund)}\left(s\right) \end{array}\right],$$

where $x_{\alpha (fund)}\left(s\right)$ is the extracted fundamental component of the signal in αβ coordinates, $x_{\alpha \beta }\left(s\right)$ is the input signal in αβ coordinates, *K* is the constant gain, and $f_{c}$ is the cut-off frequency. The value of the gain parameter has great influence on the performance of this method. Related publications [27,36] have shown that lower gain improves the accuracy of the STF, although it reduces the dynamic response, while higher gain has the opposite effect. Therefore, it is necessary to carefully choose the appropriate gain value to achieve a balance between accuracy and system dynamics. The block diagram showing the principle of this method can be seen in Figure 22.

#### 3.3.3. Enhanced PLL

**Figure 23:**
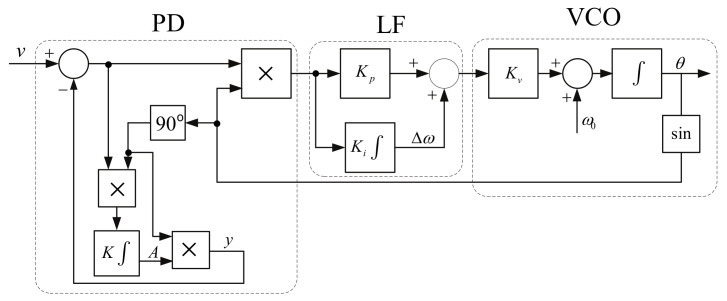
Single phase EPLL.

Enhanced PLL (EPLL) is a frequency-adaptive nonlinear synchronization approach that allows greater flexibility thanks to the introduction of the PD mechanism, which provides additional information (amplitude and phase angle) in comparison to the other PLL approaches. This method depends on the parameter *K*, which influences the speed of amplitude convergence, and the parameters $K_{p}K_{v}$ and $K_{i}K_{v}$, which influence the frequency convergence and phase rates. EPLL allows three-phase operation. The advantage of EPLL is that it maintains good robustness and is insensitive to interference from the input signal. It is usually used to synchronize grid-interfaced converters in environments with variable frequency. It can be used in single phase systems, as it provides a 90-degree shift of the input signal. The EPLL has been used in a number of studies [141,142,143]. The single phase EPLL diagram is shown in Figure 23.

#### 3.3.4. Sinusoidal Signal Integrator PLL

**Figure 24:**
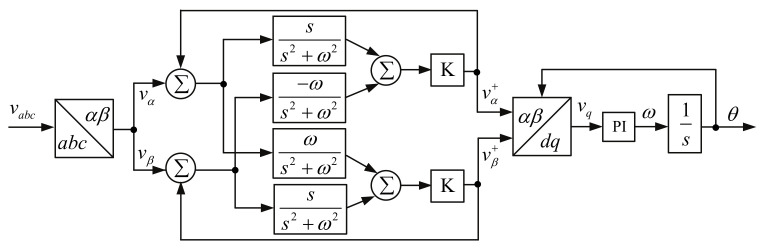
Multiple SSI-PLL.

Sinusoidal Signal Integrator PLL (SSI-PLL) extracts the fundamental positive sequence of the SF-PLL to track the utility voltage, giving it greater robustness against voltage distortions and imbalances. The *K* constant controls the bandwidth and response speed of the algorithm with multiple SSIs, as can be seen in Figure 24. In comparison, there is a different design with a single SSI, which is used as a filter for the measured utility voltage to extract the positive-sequence component $v_{\alpha }^{+}$. The $v_{\beta }^{+}$ is then calculated by delaying $v_{\alpha }^{+}$ by 90 degrees. In this design, the *K* constant controls the filtering response as well as the PLL bandwidth. Among the advantages of SSI-PLL are its immunity to voltage distortion/imbalances and the possibility of adjusting the algorithm for single-phase systems (albeit with modifications). SSI-PLL has been used in a few studies [144,145].

#### 3.3.5. Decoupled Double SRF PLL

**Figure 25:**
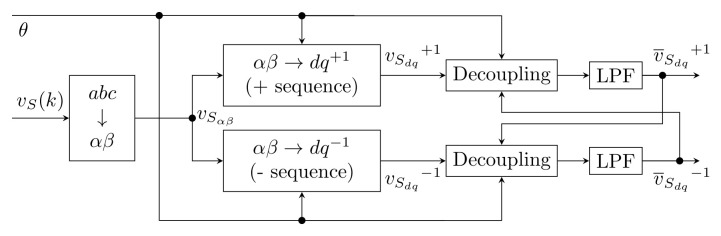
Block diagram of DDSRF-PLL method.

In contrast to the above method, this method does not work by suppressing components with a higher frequency; instead, it divides the αβ components into positive-sequence ${v_{Sdq}}^{+1}$ and negative-sequence ${v_{Sdq}}^{-1}$ components. These components are included in the signal due to an unbalanced distorted source voltage signal. Each of the components has its own SRF loop, and the separate parts of the network are subsequently processed to extract the positive-sequence ${v_{Sdq}}^{+1}$ component, which contains the basic component. Thus, the functionality is the same as for PLL (see Section 3.3). Although this method completely eliminates the problems of the basic SRF-PLL method and allows work even under significantly distorted conditions, the detection of the phase itself is a relatively complicated process due to the need for another SRF feedback loop in the control [44]. The block diagram of this method can be seen in Figure 25.

### 3.4. Conclusions of Synchronisation Techniques

In addition to the described and compared methods, there are other synchronization methods, such as the weighted least squares estimation method (WLSE), synchronized PLL based on the instantaneous real and imaginary power theory (PQ-PLL), double second order generalized integrator PLL (DSOGI-PLL), three-phase magnitude phase locked loop (3MPLL), quadrature PLL (QPLL), robust single-phase PLL (RPLL), predictive phase locked loop (PPLL), and multirate PLL (MR-PLL).

The information above suggests that the choice of synchronization method depends mostly on whether the examined grid voltage is distorted or asymmetrical. If the voltage is close to the ideal conditions, it is possible to simply use ZCD or SRF-PLL. However, this state is difficult to achieve in practical conditions. Therefore, it is usually more appropriate to choose other expansion alternatives for PLL.

Another measure by which the method can be assessed is the need to set its parameters. As stated above, all methods except for ZCD and UVC require their parameters to be set optimally in order to fulfill their required functionality. In addition, for most of them, the methodology for setting the parameters is not clearly provided. Therefore, the parameters must be set using a heuristic approach, which can be time-consuming to perform. While all methods are primarily intended for a three-phase network, many of them can be used for single-phase applications as well. A clear summary is provided in Table 2.

## 4. Further Research

Our team approached the present review mainly in the context of preparing for future research of a practical nature. This research will deal with the practical implementation of as many SAPF control methods as possible under the conditions of a real distribution network. However, the conditions of the distribution network will be fully under our control. Particular attention will be paid to maintaining the repeatability and uniformity of individual tests. This means that all individual methods will be subjected to the same conditions and the same tests. In this way, it will be possible to objectively compare all methods and quantify the “strength of the method” using a homogeneous criterion, such as the value of resulting THD${}_{I}$, relative improvement of THD${}_{I}$, Signal to Noise Ratio (SNR), or magnitude of the current flowing through the neutral wire.

Methods will be implemented on a real inverter of modular architecture of our own design, with the individual parts of SAPF able be changed independently of the others (so that it is not necessary to change the whole control system in order to change the type of synchronization algorithm). The system will therefore be of an open architecture, not be a closed solution supplied by the manufacturer. The “brain” of this system will be a control system equipped with an FPGA, upon which the individual methods will be gradually implemented and tested. The strength of the FPGA solution lies in its ability to calculate even complex computational tasks in a relatively very short period of time (on the order of units up to tens of µs), which is much shorter than one period of the fundamental frequency (20 ms, resp. 16.66 ms). Such short calculation time periods are difficult to achieve on either conventional PCs or microcontrollers. Errors caused by poor implementation or the computational delay of a slow control system can then degrade the quality of the resulting filtration, leading to the results concerning the quality of the control method being devalued. Therefore, the use of an FPGA seems to be the best solution from the point of view of testing and quantifying the filtration quality, as it introduces the smallest error into the control circuit. The primary disadvantage of this approach is the complexity of implementation, as FPGA programming is disproportionately more complex than “classic” programming (eg C, C♯, Python, etcetera).

The described algorithms can be implemented on different hardware platforms. Due to our extensive experience with National Instruments hardware, and thus with LabVIEW, we expect to be able to use multiple inhouse hardware solutions.

The first option is a combination of a PC and plug-in multifunction data acqusition board, i.e., NI PCI-6221 with 16× AI and 2× AO. The resolution of both ADC and DAC is 16 bits, and the sampling rate is up to 250 kSps. The main advantage of this platform is that the board can both generate and measure data without unnecessary buffering; this mode is called N-sample HW-timed, and allows for the shortest latency between the measured and generated values, which is equal to the reciprocal value of the sampling rate.

A second option is a combination of a PC and NI cDAQ-9185 Ethernet-connected chassis. cDAQ is a modular measurement and signal generation platform consisting of a chassis and selectable I/O modules. cDAQ-9185 is a four-slot chassis with a 2× Gb Ethernet interface. Timing and synchronization are provided by a cDAQ internal clock. cDAQ allows an N-sample method mode based on a dedicated buffer, which means that samples are read/written in blocks. The drawback of block mode is that the latency is equal to the number of samples in a block multiplied by the reciprocal value of the sampling rate. Of course, there is a certain lowest number of samples in a block.

A third option is to use a cRIO HW platform. cRIO consists of a CPU with an RT operation system, FPGA, and chassis with four or eight selectable I/O modules. The bridge between the CPU and modules is the FPGA. There are two main advantages of cRIO. First, if the code size fits into the FPGA, there is no need to calculate the samples on the CPU, as the signal processing code runs on FPGA. Code on FPGA runs natively in parallel and is extremely fast. As there is no need for buffering, the latency is the shortest of the three described methods. Second, the cRIO runs standalone, with the PC only used for parameterization or visualization.

However, the following caveat applies to all three above-mentioned methods. For continous operation, it is necessary to ensure that the computation time per sample is shorter than the inverse of the sampling rate.

The advantage of running the extraction algorithm on an FPGA lies in the near-elimination of system latency. A properly implemented harmonic extraction method on an FPGA can have the latency minimized to values as low as 4 µs. Many methods can be implemented as Sample-By-Sample processing, and therefore it is not necessary to buffer data.

A major drawback of implementing extraction algorithms on FPGAs is their very high level of difficulty from a software development perspective. The programmer cannot use floating-point data types; all functions and calculations need to be converted to a fixed-point data type.

Of course, while development can be carried out on a system that allows floating-point calculations, in which case conversion to FXP must be considered. This is the part of SW design where most problems occur, because if the conversion is not executed correctly, the data type will overflow, causing an error in the harmonic extraction method. Other problems include long compilation times and the rather complex HDL programming language.

Because this is a general synthesis of an HW circuit, it is necessary to take into account the fact that the code runs in parallel, not sequentially. This can be used, e.g., in pipelining, where several iterations of the calculation are run in parallel. However, it is necessary to program the so-called handshaking, or overhead, which decides when the calculation is valid and when it is not.

Another aim of ours is to create a database of signal voltage and current waveforms with different types of loads. Any combination of the individual waveforms will be clearly described to make it clear which scenario is to be tested (e.g., distorted line voltage THD${}_{U_{h3}}$ = 5%, THD${}_{U_{h5}}$ = 3%, non-symmetrical nature of load, thyristor-controlled rectifier RL load type XY, and others). Therefore, the individual methods can be tested on different models of the distribution network.

This database will then be freely available for download, and other authors will be able to implement their own new methods of SAPF management and objectively compare them with algorithms implemented by other authors. The indisputable advantage of this dataset will be, above all, the objectivity of the quantification of individual parameters of the filtration quality, not verbal expression of a subjective character. The key parameters we intend to examine are the convergence speed, algorithm stability, computational complexity in terms of the number of operations (addition, subtraction, multiplication, and division) in one iteration cycle, memory cell consumption, Minimum Mean Square Error (MMSE), value of the THD${}_{I}$ after filtration, relative THD${}_{I}$ improvement, and ability to function under different tested conditions.

Initial testing and implementation of the aforementioned methods will be performed in laboratory conditions. The next step will be implementation on a real “controlled” distribution network consisting of the test polygon of public SMART LED lighting on the campus of VSB (the Technical University of Ostrava) [135,146,147]. This polygon is used for comprehensive testing of LED lighting in terms of Power Quality [148], consumption, and extended functionalities (e.g., visible light communication [149,150,151,152], smart parking management [153], smart energy flow management [148], etcetera). The subject of ongoing research is primarily the impact of widespread mass deployment of smart technologies on the quality of electricity within the city distribution network. The primary advantage of this approach is that the operation of the test polygon is fully under control of the research team, making it possible to perform various tests with public lighting, even during the day when it is not normally lit. The next possible step will be to deploy the system on the distribution network of the entire campus, thus contributing to the ongoing project to create a small “SMART city” on the university grounds (SMART street lighting, SMART information boards, SMART energy flow management, etcetera) or deployment to one of the industrial companies of our team’s cooperating industrial partners.

## 5. Conclusions

It is quite obvious that current harmonics are one of the most important elements that fundamentally effect the power quality of electricity within a distribution grid. Great efforts are currently being made to compensate for these higher current components. One of the most effective methods of solving this problem of suppression of higher harmonic components is the deployment of SAPF. Their key feature is that their efficiency is directly reflected in the speed and accuracy of control algorithms. Due to the massive deployment of dynamically changing systems (from a higher harmonic content perspective), it is necessary to guarantee a high degree of flexibility in terms of individual operating scenarios. These scenarios are covered by modern control algorithms thanks to the use of progressive signal processing methods. The development of this area is evoked by the availability of powerful and affordable semiconductor switching elements.

This research has systematically summarized and compared existing SAPF control methods, with an emphasis on modern methods of signal processing which are beginning to be significantly applied in the field of power engineering. Thanks to the relatively easy availability of FPGAs, it is possible to implement complex control algorithms which, in combination with new semiconductor switching elements, are able to achieve high efficiency.

This publication should help to understand the basic principle of SAPF while providing a subjective classification and comparison of different control algorithms that can serve as a basis for further research in this progressing field.

## Figures and Tables

**Table 1:** Summary of Harmonic Extraction Methods.

Algorithm	One-Phase	Three-Phase	Distorted Conditions	Only Current Meas.	No Need to Set Parameters	Implementation Complexity (1–5)	Advantages	Disadvantages
**iPQ**	x	✓	x	x	✓	1	Easy to implement and correct functionality with balanced and sinusoidal voltage.	Necessary to measure three voltages and currents. Impaired function in case of unbalanced and non-sinusoidal voltages.
**CVT**	x	✓	x	x	✓	1	Easy to implement and correct functionality with balanced and sinusoidal voltage. Eliminates neutral current in all cases.	Same as iPQ.
**PQR**	x	✓	x	x	✓	2	Easy to implement and correct functionality with balanced and sinusoidal voltage. Eliminates neutral current in all cases.	Two transformations are needed.
**UPC**	x	✓	x	x	✓	1	The power factor always has a value of 1, so the instantaneous reactive power is eliminated.	Method does not meet IEEE 1459 standard, and the current waveforms correspond to voltage, thus it may not correspond to IEEE 519 standard.
**PHC**	x	✓	✓	x	✓	1	Method is insensitive to external negative conditions such as distorted or imbalanced main voltage. Eliminates neutral current in all cases.	Necessary to measure three voltages and currents. The power factor does not take the value of 1.
**SRF**	x	✓	✓	✓	✓	2	It is not necessary to measure voltage. This method is simple to implement and often used.	Cannot be used for single-phase system.
**SDM**	x	✓	✓	x	✓	2	Works effectively in both balanced and unbalanced systems. Eliminates neutral current in all cases.	Significantly slower than iPQ. It is necessary to measure three voltages and currents.
**CCT**	✓	✓	✓	x	✓	2	Method does not require any transformation, and all calculations are performed in a-b-c coordinate system.	Necessary to measure three voltages and currents.
**SMT**	✓	✓	✓	x	✓	2	Works effectively in both balanced and unbalanced system. Eliminates neutral current in all cases.	Necessary to measure three voltages and currents.
**VF**	x	✓	✓	x	✓	2	Method is insensitive to external negative conditions such as distorted or imbalanced main voltage. Eliminates neutral current in all cases.	Necessary to measure three voltages and currents.
**CPT**	✓	✓	✓	x	✓	2	Method does not require any transformation and all calculations are performed in a-b-c coordinate system. abc.	Necessary to measure three voltages and currents. Computationally demanding.
**FFT & DFT**	✓	✓	✓	✓	x	2	Easy to implement.	Necessary to measure a whole period for correct estimation of the reference current. FFT and DFT are sensitive to incomplete periods.
**RDFT**	✓	✓	✓	✓	x	3	Method calculates the reference current from N samples and does not require whole period. Suitable for real-time applications. Less computationally demanding than FFT and DFT.	Necessary to measure a whole period for correct estimation of the reference current. RDFT is sensitive to incomplete periods.
**WT**	✓	✓	✓	✓	x	3	Easy to implement.	Necessary to measure at least one whole measuring window $T_{W}$.
**HT**	✓	✓	✓	✓	✓	2	Easy to implement. Better than FFT in case of noisy signals.	Not able to determine short-time and weak disturbances.
**KF**	✓	✓	✓	✓	x	3	Easy to implement and computationally undemanding.	Method assumes that the system and observation models are linear, which does not correspond to real systems.
**EKF**	✓	✓	✓	✓	x	3	Easy to implement and computationally undemanding. Can be used in nonlinear systems.	Filter parameters dependent on measured input data.
**ADALINE**	✓	✓	✓	✓	x	5	The results of filtration are independent of external conditions and the method manages to filter even dynamically changing types of distortion.	High computational complexity. Sensitive to bad settings. Strongly heuristic.
**ANFIS**	✓	✓	✓	✓	x	5	The results of filtration are independent of external conditions and the method manages to filter even dynamically changing types of distortion.	High computational complexity. Sensitive to bad settings. Strongly heuristic.
**LMS**	✓	✓	✓	✓	x	3	Easy to implement.	Necessary to choose correct settings of the adaptive filter. Time of convergence is longer than RLS.
**RLS**	✓	✓	✓	✓	x	4	Easier setup of the adaptive algorithm than LMS. Time of convergence is shorter than LMS.	More complex implementation than LMS.
**Notch LMS**	x	✓	✓	✓	x	4	The filter is used only as second order, so the delay is one sample. Suitable for FPGA implementation.	Strongly dependent on the setting of the µ parameter.
**Notch RLS**	x	✓	✓	✓	x	4	The filter is used only as second order, so the delay is one sample. Suitable for FPGA implementation.	More complex implementation than Notch LMS.

**Table 2:** Summary of mentioned synchronisation techniques.

Technique	One-Phase	Three-Phase	Non-Ideal Voltage Conditions	Heuristic Setting	Implementation Complexity (1–5)	Advantages	Disadvantages
**ZCD**	✓	✓	x	x	1	Easy to control.	HW circuit, noise sensitive, slow response to dynamic changes, poor results under distorted network conditions.
**SV**	x	✓	x	x	3	Robust and not sensitive to disturbances. If configured properly, it provides highly distortion-free estimation.	Sensitivity to the input frequency variation and imbalance.
**EPLL**	x	✓	✓	x	3	Good robustness. Insensitivity to inferences from the input signal. Used to synchronize grid-interfaced converters. Provides additional information regarding amplitude and phase angle due to introduction of PD.	Requires correct setting of the *K* parameter.
**SSI-PLL**	✓	✓	✓	✓	4	Immunity to voltage distortion and imbalance. Adaptable.	Requires correct setting of the filtering response and bandwidth parameters.
**SRF-PLL**	✓	✓	x	✓	2	Easy to implement, accurate synchronization under ideal network conditions, one of the most commonly used methods.	The PI controller must be optimally set, and it cannot work under distorted network conditions.
**STF-PLL**	✓	✓	✓	✓	3	Suitable for implementation under distorted or asymmetric conditions.	PI controller must be optimally set, STF gain must be chosen carefully, STF integration makes control more complicated.
**DDSRF-PLL**	x	✓	✓	✓	3	Suitable for implementation under distorted or asymmetric conditions.	The PI controller must be optimally set, and the extra SRF loop increases the computational complexity of the method.

## Data Availability

Not applicable.

## Abbreviations

The following abbreviations are used in this manuscript:

ANFIS	Adaptive Neuro-Fuzzy Inference System
DCC	Direct Current Control
DFT	Discrete Fourier Transform
DSP	Digital Signal Processor
FCE	Fundamental Component Extraction
FFT	Fast Fourier Transformation
FIR	Finite Impulse Response
FIS	Fuzzy Inference System
FPGA	Field Programmable Gate Array
GD	Gradient Descent
GTO	Gate turn-off
HPF	High-Pass Filter
ICC	Indirect Current Control
IGBT	Insulated Gate Bipolar transistors
IGCT	Integrated gate commutated
IIR	Infinite Impulse Response
LF	Loop Filter
LMS	Least Mean Squares
LPF	Low-Pass Filter
MMSE	Minimum Mean Square Error
MNN	Multilayer Neural Network
PCC	Point of Common Coupling
PD	Phase Detector
PLL	Phase-Locked Loop
PWM	Pulse-Width Modulation
RDFT	Recursive Discrete Fourier Transform
RLS	Recursive Least Squares
SAPF	Shunt-Active Power Filter
SCG	Scale Conjugate Gradient
SNR	Signal to Noise Ratio
SVPWM	Space Vector PWM
THD	Total Harmonic Distortion
VCO	Voltage-Controlled Oscillator
ZCD	Zero-Cross Detection

## References

[1] Thakur P. (2020). Load Distribution and VFD Topology Selection for Harmonic Mitigation in an Optimal Way. IEEE Trans. Ind. Appl..

[2] Xu Y., Xiao X., Liu H., Wang H. Parallel Operation of Hybrid Active Power Filter with Passive Power Filter or Capacitors. Proceedings of the 2005 IEEE/PES Transmission & Distribution Conference & Exposition: Asia and Pacific.

[3] Arritt R.F., Dugan R.C. Distributed Generation Interconnection Transformer and Grounding Selection. Proceedings of the 2008 IEEE Power and Energy Society General Meeting—Conversion and Delivery of Electrical Energy in the 21st Century.

[4] Baghzouz Y., Gong X. (1995). Analysis of Three-Phase Transformer No-Load Characteristics. IEEE Trans. Power Syst..

[5] Sanjay J.S., Misra B. Power Quality Improvement for Non Linear Load Applications Using Passive Filters. Proceedings of the 2019 3rd International Conference on Recent Developments in Control, Automation & Power Engineering (RDCAPE).

[6] Phannil N., Jettanasen C., Ngaopitakkul A. (2018). Harmonics and Reduction of Energy Consumption in Lighting Systems by Using LED Lamps. Energies.

[7] Goschler C. (2017). Compensation in Practice: The Foundation ‘Remembrance, Responsibility and Future’ and the Legacy of Forced Labour during the Third Reich.

[8] Hansen S., Nielsen P., Blaabjerg F. (2000). Harmonic Cancellation by Mixing Nonlinear Single-Phase and Three-Phase Loads. IEEE Trans. Ind. Appl..

[9] Rastogi M., Mohan N., Edris A.A. (1995). Hybrid-Active Filtering of Harmonic Currents in Power Systems. IEEE Trans. Power Deliv..

[10] Akagi H. (2005). Active Harmonic Filters. Proc. IEEE.

[11] Duda T. (2015). Robust Algorithms for Control of Dynamic Systems. Ph.D. Thesis.

[12] Chamrád P. (2019). Optimalization of Parameters of MRAS Observer for Sensorless Control of Asynchronal Motor. Ph.D. Thesis.

[13] Massoud A., Finney S., Williams B. Review of Harmonic Current Extraction Techniques for an Active Power Filter. Proceedings of the 2004 11th International Conference on Harmonics and Quality of Power (IEEE Cat. No.04EX951).

[14] Abu Hasim A.S., Talib M.H.N., Ibrahim Z. Comparative Study of Different PWM Control Scheme for Three-Phase Three-Wire Shunt Active Power Filter. Proceedings of the 2012 IEEE International Power Engineering and Optimization Conference.

[15] Kim H., Blaabjerg F., Bak-Jensen B., Choi J. Instantaneous Power Compensation in Three-Phase Systems by Using p-q-r Theory. Proceedings of the 2001 IEEE 32nd Annual Power Electronics Specialists Conference (IEEE Cat. No. 01CH37230).

[16] Montero M.I.M., Cadaval E.R., Gonzalez F.B. (2007). Comparison of Control Strategies for Shunt Active Power Filters in Three-Phase Four-Wire Systems. IEEE Trans. Power Electron..

[17] Herrera R.S., Salmerón P., Kim H. (2008). Instantaneous Reactive Power Theory Applied to Active Power Filter Compensation: Different Approaches, Assessment, and Experimental Results. IEEE Trans. Ind. Electron..

[18] Benhabib M., Saadate S. (2005). New Control Approach for Four-Wire Active Power Filter Based on the Use of Synchronous Reference Frame. Electr. Power Syst. Res..

[19] Revuelta P.S., Herrera R.S. Application of the Instantaneous Power Theories in Load Compensation with Active Power Filters. Proceedings of the 10th European Conference on Power Electronics and Applications.

[20] Salam Z., Tan P.C., Jusoh A. (2006). Harmonics Mitigation Using Active Power Filter: A Technological Review. Elektr. J. Electr. Eng..

[21] Dolen M., Lorenz R. An Industrially Useful Means for Decomposition and Differentiation of Harmonic Components of Periodic Waveforms. Proceedings of the Conference Record of the 2000 IEEE Industry Applications Conference. Thirty-Fifth IAS Annual Meeting and World Conference on Industrial Applications of Electrical Energy (Cat. No.00CH37129).

[22] Mortezaei A., Lute C., Simoes M.G., Marafao F.P., Boglia A. PQ, DQ and CPT Control Methods for Shunt Active Compensators—A Comparative Study. Proceedings of the 2014 IEEE Energy Conversion Congress and Exposition (ECCE).

[23] Qasim M., Khadkikar V. (2014). Application of Artificial Neural Networks for Shunt Active Power Filter Control. IEEE Trans. Ind. Inform..

[24] Routhu B., Arun N. (2013). PI, FUZZY and ANFIS Control of 3-Phase Shunt Active Power Filter. Int. J. Eng. Technol. (IJET).

[25] Tamboli D.A., Chile R.H. Reference Signal Generation for Shunt Active Power Filter Using Adaptive Filtering Approach. Proceedings of the 2015 International Conference on Industrial Instrumentation and Control (ICIC).

[26] Abdeslam D.O., Wira P., Merckle J., Flieller D., Chapuis Y.A. (2007). A Unified Artificial Neural Network Architecture for Active Power Filters. IEEE Trans. Ind. Electron..

[27] Abdusalam M., Poure P., Karimi S., Saadate S. (2009). New Digital Reference Current Generation for Shunt Active Power Filter under Distorted Voltage Conditions. Electr. Power Syst. Res..

[28] Bacon V.D., de Souza V., Padim E.T., da Silva S.A.O. Influence of the PLL Phase-Angle Quality on the Static and Dynamic Performance of Grid-Connected Systems. Proceedings of the 2017 Brazilian Power Electronics Conference (COBEP).

[29] Bhattacharya A., Chakraborty C. (2011). A Shunt Active Power Filter With Enhanced Performance Using ANN-Based Predictive and Adaptive Controllers. IEEE Trans. Ind. Electron..

[30] Blaabjerg F., Teodorescu R., Liserre M., Timbus A. (2006). Overview of Control and Grid Synchronization for Distributed Power Generation Systems. IEEE Trans. Ind. Electron..

[31] Da Silva S.A.O., Bacon V.D., Campanhol L.B.G., Angélico B.A. An Adaptive Phase-Locked Loop Algorithm for Single-Phase Utility Connected System. Proceedings of the 2013 15th European Conference on Power Electronics and Applications (EPE).

[32] Djazia K., Krim F., Chaoui A., Sarra M. (2015). Active Power Filtering Using the ZDPC Method under Unbalanced and Distorted Grid Voltage Conditions. Energies.

[33] Elangovan S., Thanushkodi K., Neelakantan P.N. (2014). A Two Level Shunt Active Power Filter without Pll for Industrial Loads. Aust. J. Basic Appl. Sci.

[34] Golestan S., Monfared M., Freijedo F.D. (2013). Design-Oriented Study of Advanced Synchronous Reference Frame Phase-Locked Loops. IEEE Trans. Power Electron..

[35] Han Y., Xu L., Khan M.M., Yao G., Zhou L.D., Chen C. (2009). A Novel Synchronization Scheme for Grid-Connected Converters by Using Adaptive Linear Optimal Filter Based PLL (ALOF–PLL). Simul. Model. Pract. Theory.

[36] Hoon Y., Radzi M.A.M., Hassan M.K., Mailah N.F. (2018). Operation of Three-Level Inverter-Based Shunt Active Power Filter Under Nonideal Grid Voltage Conditions With Dual Fundamental Component Extraction. IEEE Trans. Power Electron..

[37] Jaalam N., Rahim N., Bakar A., Tan C., Haidar A.M. (2016). A Comprehensive Review of Synchronization Methods for Grid-Connected Converters of Renewable Energy Source. Renew. Sustain. Energy Rev..

[38] McGrath B., Holmes D., Galloway Galloway J. (2005). Power Converter Line Synchronization Using a Discrete Fourier Transform (DFT) Based on a Variable Sample Rate. IEEE Trans. Power Electron..

[39] Mohd Zainuri M., Mohd Radzi M., Che Soh A., Mariun N., Abd Rahim N., Hajighorbani S. (2016). Fundamental Active Current Adaptive Linear Neural Networks for Photovoltaic Shunt Active Power Filters. Energies.

[40] Oliveira da Silva S.A., Garcia Campanhol L.B., Goedtel A. (2014). Application of Shunt Active Power Filter for Harmonic Reduction and Reactive Power Compensation in Three-Phase Four-Wire Systems. IET Power Electron..

[41] Özerdem Ö.C., Khadem S.K., Biricik S., Basu M., Redif S. (2014). Real-Time Control of Shunt Active Power Filter under Distorted Grid Voltage and Unbalanced Load Condition Using Self-Tuning Filter. IET Power Electron..

[42] Radzi M., Rahim N. (2009). Neural Network and Bandless Hysteresis Approach to Control Switched Capacitor Active Power Filter for Reduction of Harmonics. IEEE Trans. Ind. Electron..

[43] Rahman N.F.A., Radzi M.A.M., Soh A.C., Mariun N., Rahim N.A. (2015). Dual Function of Unified Adaptive Linear Neurons Based Fundamental Component Extraction Algorithm for Shunt Active Power Filter Operation. Int. Rev. Electr. Eng..

[44] Rodriguez P., Pou J., Bergas J., Candela J.I., Burgos R.P., Boroyevich D. (2007). Decoupled Double Synchronous Reference Frame PLL for Power Converters Control. IEEE Trans. Power Electron..

[45] Shah M.C., Chauhan S.K., Tekwani P.N., Tiwari R.R. (2014). Analysis, Design and Digital Implementation of a Shunt Active Power Filter with Different Schemes of Reference Current Generation. IET Power Electron..

[46] Shinnaka S. (2002). A New Characteristics-Variable Two-Input/Output Filter in D-Module-Designs, Realizations, and Equivalence. IEEE Trans. Ind. Appl..

[47] Timbus A., Liserre M., Teodorescu R., Blaabjerg F. Synchronization Methods for Three Phase Distributed Power Generation Systems. An Overview and Evaluation. Proceedings of the IEEE 36th Conference on Power Electronics Specialists.

[48] Vainio O., Ovaska S., Polla M. (2003). Adaptive Filtering Using Multiplicative General Parameters for Zero-Crossing Detection. IEEE Trans. Ind. Electron..

[49] Hoon Y., Mohd Radzi M.A., Hassan M.K., Mailah N.F. (2017). A Self-Tuning Filter-Based Adaptive Linear Neuron Approach for Operation of Three-Level Inverter-Based Shunt Active Power Filters under Non-Ideal Source Voltage Conditions. Energies.

[50] Zainuri M., Radzi M.M., Soh A.C., Mariun N., Rahim N.A. (2016). Simplified Adaptive Linear Neuron Harmonics Extraction Algorithm for Dynamic Performance of Shunt Active Power Filter. Int. Rev. Model. Simul..

[51] Girgis A., Chang W., Makram E. (1991). A Digital Recursive Measurement Scheme for Online Tracking of Power System Harmonics. IEEE Trans. Power Deliv..

[52] Ribeiro R.L.A., Rocha T.O.A., de Sousa R.M., dos Santos E.C., Lima A.M.N. (2015). A Robust DC-Link Voltage Control Strategy to Enhance the Performance of Shunt Active Power Filters Without Harmonic Detection Schemes. IEEE Trans. Ind. Electron..

[53] Kumar S.J., Sangeetha P., Charan C.R. Shunt Active Power Filter Control by Instantaneous Reactive Power Compensation. Proceedings of the 2016 International Conference on Signal Processing, Communication, Power and Embedded System (SCOPES).

[54] Geetha K., Sangeetha B. Performance Evaluation of Conventional and Intelligent Controller Based Shunt Active Filter. Proceedings of the Third International Conference on Computational Intelligence and Information Technology (CIIT 2013).

[55] Patjoshi R.K., Mahapatra K.K. Performance Comparison of Direct and Indirect Current Control Techniques Applied to a Sliding Mode Based Shunt Active Power Filter. Proceedings of the 2013 Annual IEEE India Conference (INDICON).

[56] Rahman N.A., Nasir N.M., Baharom R. Comparative Study of Direct and Indirect Current Control Algorithms for Shunt Active Power Filter. Proceedings of the 2019 International Conference on Electrical Engineering and Informatics (ICEEI).

[57] Imad A., Hani S.E., Mediouni H., Echchaachouai A. Comparative Analysis on Current Control Methods of Shunt Active Power Filter for the Improvement of Grid Energy Quality. Proceedings of the 2017 International Conference on Electrical and Information Technologies (ICEIT).

[58] Akagi H., Kanazawa Y., Nabae A. (1984). Instantaneous Reactive Power Compensators Comprising Switching Devices without Energy Storage Components. IEEE Trans. Ind. Appl..

[59] Peng F.Z., Lai J.S. (1996). Generalized Instantaneous Reactive Power Theory for Three-Phase Power Systems. IEEE Trans. Instrum. Meas..

[60] Nabae A., Tanaka T. (1996). A New Definition of Instantaneous Active-Reactive Current and Power Based on Instantaneous Space Vectors on Polar Coordinates in Three-Phase Circuits. IEEE Trans. Power Deliv..

[61] Fujita H., Akagi H. (1998). The Unified Power Quality Conditioner: The Integration of Series-and Shunt-Active Filters. IEEE Trans. Power Electron..

[62] Peng F.Z., Ott G.W., Adams D.J. (1998). Harmonic and Reactive Power Compensation Based on the Generalized Instantaneous Reactive Power Theory for Three-Phase Four-Wire Systems. IEEE Trans. Power Electron..

[63] Ghosh A., Joshi A. (2000). A New Approach to Load Balancing and Power Factor Correction in Power Distribution System. IEEE Trans. Power Deliv..

[64] Boussaid A., Nemmour A.L., Louze L., Khezzar A. (2015). A Novel Strategy for Shunt Active Filter Control. Electr. Power Syst. Res..

[65] Büyük M., İnci M., Tan A., Tümay M. (2019). Improved Instantaneous Power Theory Based Current Harmonic Extraction for Unbalanced Electrical Grid Conditions. Electr. Power Syst. Res..

[66] Kim H., Akagi H. The Instantaneous Power Theory on the Rotating P-q-r Reference Frames. Proceedings of the IEEE 1999 International Conference on Power Electronics and Drive Systems. PEDS’99 (Cat. No. 99TH8475).

[67] Chebabhi A., Fellah M.K., Benkhoris M.F. (2015). Application of PQR Theory for Control of a 3-Phase 4-Wire 4-Legs Shunt Active Power Filter in the *αβ*o-Axes Using 3d-SVM Technique. Leonardo J. Sci..

[68] Rafiei S.R., Toliyat H., Ghazi R., Gopalarathnam T. (2001). An Optimal and Flexible Control Strategy for Active Filtering and Power Factor Correction under Non-Sinusoidal Line Voltages. IEEE Trans. Power Deliv..

[69] Zhao H.J., Pang Y.F., Qiu Z.M., Chen M. (2006). Study on UPF Harmonic Current Detection Method Based on DSP. J. Phys. Conf. Ser..

[70] Eid A., Abdel-Salam M., El-Kishky H., El-Mohandes T. (2009). Active Power Filters for Harmonic Cancellation in Conventional and Advanced Aircraft Electric Power Systems. Electr. Power Syst. Res..

[71] Bhattacharya S., Frank T.M., Divan D.M., Banerjee B. (1998). Active Filter System Implementation. IEEE Ind. Appl. Mag..

[72] Bhattacharya S., Divan D. Synchronous Frame Based Controller Implementation for a Hybrid Series Active Filter System. Proceedings of the In IAS’95. Conference Record of the 1995 IEEE Industry Applications Conference Thirtieth IAS Annual Meeting.

[73] Da Silva C.H., Pereira R.R., da Silva L.E.B., Lambert-Torres G., Bose B.K., Ahn S.U. (2010). A Digital PLL Scheme for Three-Phase System Using Modified Synchronous Reference Frame. IEEE Trans. Ind. Electron..

[74] Hoon Y., Radzi M.A.M., Hassan M.K., Mailah N.F. Three-Phase Three-Level Shunt Active Power Filter with Simplified Synchronous Reference Frame. Proceedings of the 2016 IEEE Industrial Electronics and Applications Conference (IEACon).

[75] Hoon Y., Radzi M.A.M., Hassan M.K., Mailah N.F., Wahab N.I.A. (2016). A Simplified Synchronous Reference Frame for Indirect Current Controlled Three-Level Inverter-Based Shunt Active Power Filters. J. Power Electron..

[76] Kumar R., Bansal H.O. (2019). Real-Time Implementation of Adaptive PV-Integrated SAPF to Enhance Power Quality. Int. Trans. Electr. Energy Syst..

[77] Msigwa C.J., Kundy B.J., Mwinyiwiwa B.M. (2009). Control Algorithm for Shunt Active Power Filter Using Synchronous Reference Frame Theory. World Acad. Sci. Eng. Technol..

[78] Musa S., Radzi M.A.M., Hizam H., Wahab N.I.A., Hoon Y., Zainuri M.A.A.M. (2017). Modified Synchronous Reference Frame Based Shunt Active Power Filter with Fuzzy Logic Control Pulse Width Modulation Inverter. Energies.

[79] Pigazo A., Moreno V.M., Estebanez E.J. (2009). A Recursive Park Transformation to Improve the Performance of Synchronous Reference Frame Controllers in Shunt Active Power Filters. IEEE Trans. Power Electron..

[80] Sun B., Dai N.Y., Chio U.F., Wong M.C., Wong C.K., Sin S.W., Seng-Pan U., Martins R.P. FPGA-Based Decoupled Double Synchronous Reference Frame PLL for Active Power Filters. Proceedings of the 2011 6th IEEE Conference on Industrial Electronics and Applications.

[81] Sundaram E., Venugopal M. (2016). On Design and Implementation of Three Phase Three Level Shunt Active Power Filter for Harmonic Reduction Using Synchronous Reference Frame Theory. Int. J. Electr. Power Energy Syst..

[82] Mattavelli P. (2001). Synchronous-Frame Harmonic Control for High-Performance AC Power Supplies. IEEE Trans. Ind. Appl..

[83] Escobar G., Stankovic A.M., Mattavelli P. (2004). An Adaptive Controller in Stationary Reference Frame for D-Statcom in Unbalanced Operation. IEEE Trans. Ind. Electron..

[84] Marmouh S., Boutoubat M., Mokrani L. (2018). Performance and Power Quality Improvement Based on DC-Bus Voltage Regulation of a Stand-Alone Hybrid Energy System. Electr. Power Syst. Res..

[85] De Lacerda de Oliveira L., da Silva L., da Silva V., Torres G., Pinto J. Improving the Dynamic Response of Active Power Filters Based on the Synchronous Reference Frame Method. Proceedings of the APEC, Seventeenth Annual IEEE Applied Power Electronics Conference and Exposition (Cat. No. 02CH37335).

[86] Kabir M.A., Mahbub U. Synchronous Detection and Digital Control of Shunt Active Power Filter in Power Quality Improvement. Proceedings of the 2011 IEEE Power and Energy Conference at Illinois.

[87] Bajaj M., Rautela S., Sharma A. A Comparative Analysis of Control Techniques of SAPF under Source Side Disturbance. Proceedings of the In 2016 International Conference on Circuit, Power and Computing Technologies (ICCPCT).

[88] Narongrit T., Santiprapan P., Janpong S. A Synchronous Detection with Fourier Analysis for Single-Phase Shunt Active Power Filters. Proceedings of the 2018 5th International Conference on Electric Power and Energy Conversion Systems (EPECS).

[89] Tali M., Essadki A., Nasser T. Harmonic Detection Methods of Shunt Active Power Filter under Unbalanced Loads. Proceedings of the 2016 International Renewable and Sustainable Energy Conference (IRSEC).

[90] Sujatha C.H., Kusam S., Shekar K.C. (2011). Shunt Active Filter Algorithms for a Three Phase System Fed to Adjustable Speed Drive. Int. J. Eng. Sci. Technol..

[91] Tanaka T., Okamoto M., Hiraki E. Control Strategies of Active Power Line Conditioners in Single-Phase Circuits. Proceedings of the 8th International Conference on Power Electronics—ECCE Asia.

[92] Dongre G.A., Choudhari V.V., Diwan S.P. A Comparison and Analysis of Control Algorithms for Shunt Active Power Filter. Proceedings of the 2015 International Conference on Computation of Power, Energy, Information and Communication (ICCPEIC).

[93] Jou H.L. (1994). New Single-Phase Active Power Filter. IEE Proc.-Electr. Power Appl..

[94] Hsu C., Wu H. (1996). A New Single-Phase Active Power Filter with Reduced Energy-Storage Capacity. IEE Proc.-Electr. Power Appl..

[95] Bains B.K., Dhingra A. (2018). A Review of Current Control Techniques for Active Power Filter Applications. J. Eng. Econ. Dev..

[96] Tenti P., Mattavelli P., Morales Paredes H.K. Conservative Power Theory, Sequence Components and Accountability in Smart Grids. Proceedings of the 2010 International School on Nonsinusoidal Currents and Compensation.

[97] Rosa R.B., Vahedi H., Godoy R.B., Pinto J.O.P., Al-Haddad K. Conservative Power Theory Used in NPC-Based Shunt Active Power Filter to Eliminate Electric Metro System Harmonics. Proceedings of the 2015 IEEE Vehicle Power and Propulsion Conference (VPPC).

[98] Haugan T.S., Tedeschi E. Reactive and Harmonic Compensation Using the Conservative Power Theory. Proceedings of the 2015 Tenth International Conference on Ecological Vehicles and Renewable Energies (EVER).

[99] Taher S.A., Alaee M.H., Dehghani Arani Z. Model Predictive Control of PV-Based Shunt Active Power Filter in Single Phase Low Voltage Grid Using Conservative Power Theory. Proceedings of the 2017 8th Power Electronics, Drive Systems & Technologies Conference (PEDSTC).

[100] Bitoleanu A., Popescu M. Shunt Active Power Filter Overview on the Reference Current Methods Calculation and Their Implementation. Proceedings of the 2013 4th International Symposium on Electrical and Electronics Engineering (ISEEE).

[101] Paredes H.K.M., Brandao D.I., Terrazas T.M., Marafao F.P. Shunt Active Compensation Based on the Conservative Power Theory Current’s Decomposition. Proceedings of the XI Brazilian Power Electronics Conference.

[102] Suru C.V., Patrascu A., Linca M. Conservative Power Theory Implementation in Shunt Active Power Filtering. Proceedings of the International School on Nonsinusoidal Currents and Compensation 2013 (ISNCC 2013).

[103] Karbasforooshan M.S., Monfared M. An Adaptive Recursive Discrete Fourier Transform Technique for the Reference Current Generation of Single-Phase Shunt Active Power Filters. Proceedings of the 2016 7th Power Electronics and Drive Systems Technologies Conference (PEDSTC).

[104] Borisov K., Ginn H., Chen G. (2009). A Computationally Efficient RDFT-Based Reference Signal Generator for Active Compensators. IEEE Trans. Power Deliv..

[105] Reza M.S., Ciobotaru M., Agelidis V.G. A Recursive DFT Based Technique for Accurate Estimation of Grid Voltage Frequency. Proceedings of the IECON 2013—39th Annual Conference of the IEEE Industrial Electronics Society.

[106] Asiminoael L., Blaabjerg F., Hansen S. (2007). Detection Is Key—Harmonic Detection Methods for Active Power Filter Applications. IEEE Ind. Appl. Mag..

[107] Ginn H.L., Chen G. (2014). Digital Control Method for Grid-Connected Converters Supplied With Nonideal Voltage. IEEE Trans. Ind. Inform..

[108] Kumar R., Bansal H.O. (2019). Hardware in the Loop Implementation of Wavelet Based Strategy in Shunt Active Power Filter to Mitigate Power Quality Issues. Electr. Power Syst. Res..

[109] Firouzjah K.G., Sheikholeslami A., Karami-Mollaei M.R., Khaleghi M. (2008). A New Harmonic Detection Method for Shunt Active Filter Based on Wavelet Transform. J. Appl. Sci. Res..

[110] Driesen J., Belmans R. Active Power Filter Control Algorithms Using Wavelet-Based Power Definitions. Proceedings of the 10th International Conference on Harmonics and Quality of Power. Proceedings (Cat. No. 02EX630).

[111] Aghazadeh A., Niazazari I., Khodabakhshi N., Hosseinian S.H., Abyaneh H.A. A New Method of Single-Phase Active Power Filter for AC Electric Railway System Based on Hilbert Transform. Proceedings of the 2013 IEEE International Conference on Smart Energy Grid Engineering (SEGE).

[112] Swarnkar N.K., Mahela O.P., Lalwani M. Evaluation of Power Quality in Distribution System with High Penetration of Wind Power Generation. Proceedings of the 2021 Innovations in Power and Advanced Computing Technologies (i-PACT).

[113] Panigrahi R., Subudhi B. (2017). Performance Enhancement of Shunt Active Power Filter Using a Kalman Filter-Based H*∞* Control Strategy. IEEE Trans. Power Electron..

[114] Panigrahi R., Panda P.C., Subudhi B.D. New Strategy for Generation of Reference Current in Active Power Filters with Distortion in Line Voltage. Proceedings of the 2012 IEEE 7th International Conference on Industrial and Information Systems (ICIIS).

[115] Hasim A.S.B.A., Dardin S.M.F.B.S.M., Ibrahim Z.B., Serra G.L. (2018). Kalman Filters for Reference Current Generation in Shunt Active Power Filter (APF). Kalman Filters—Theory for Advanced Applications.

[116] Prince S.K., Panda K.P., Kumar V.N., Panda G. Power Quality Enhancement in a Distribution Network Using PSO Assisted Kalman Filter—Based Shunt Active Power Filter. Proceedings of the 2018 IEEMA Engineer Infinite Conference (eTechNxT).

[117] Hoffmann N., Fuchs F.W. (2014). Minimal Invasive Equivalent Grid Impedance Estimation in Inductive—Resistive Power Networks Using Extended Kalman Filter. IEEE Trans. Power Electron..

[118] Panigrahi R., Subudhi B., Panda P.C. (2015). Model Predictive-based Shunt Active Power Filter with a New Reference Current Estimation Strategy. IET Power Electron..

[119] Panigrahi R., Patjoshi R.K. (2020). Robust Extended Complex Kalman Filter Based LQR Control Strategy of Shunt Active Power Filter. Int. J. Electr. Eng. Inform..

[120] Prince S.K., Panda K.P., Patowary M., Panda G. FPA Tuned Extended Kalman Filter for Power Quality Enhancement in PV Integrated Shunt Active Power Filter. Proceedings of the 2019 International Conference on Computing, Power and Communication Technologies (GUCON).

[121] Regulski P., Terzija V. (2012). Estimation of Frequency and Fundamental Power Components Using an Unscented Kalman Filter. IEEE Trans. Instrum. Meas..

[122] Anderson J.A., Rosenfeld E. (2000). Talking Nets: An Oral History of Neural Networks.

[123] Dash P., Swain D., Liew A., Rahman S. (1996). An Adaptive Linear Combiner for On-Line Tracking of Power System Harmonics. IEEE Trans. Power Syst..

[124] Hammer M., Janda O., Ertl J. (2012). Selected Soft-Computing Methods in Power Oil Transformer Diagnostics—Part 1. J. Elektrorevue.

[125] Martinek R., Žídek J. (2012). Refining the Diagnostic Quality of the Abdominal Fetal Electrocardiogram Using the Techniques of Artificial Intelligence. Prz. Elektrotech..

[126] Shukla A., Tiwari R., Kala R. (2010). Towards Hybrid and Adaptive Computing.

[127] Karthikeyan V.V., Kalpana M. (2013). Power Quality Enhancement Using Shunt Active Filter with ANFIS Controller. Int. J. Adv. Inf. Sci. Technol..

[128] Martinek R., Manas J., Zidek J., Bilik P. (2013). Power Quality Improvement by Shunt Active Performance Filters Emulated by Artificial Intelligence Techniques. Proceedings of the 2nd International Conference on Advances in Computer Science and Engineering.

[129] Terriche Y., Guerrero J.M., Vasquez J.C. (2018). Performance Improvement of Shunt Active Power Filter Based on Non-Linear Least-Square Approach. Electr. Power Syst. Res..

[130] Martinek R., Zidek J., Bilik P., Manas J., Koziorek J., Teng Z., Wen H. (2013). The Use of LMS and RLS Adaptive Algorithms for an Adaptive Control Method of Active Power Filter. Energy Power Eng..

[131] Clarkson P.M. (2017). Optimal and Adaptive Signal Processing.

[132] Martinek R., Rzidky J., Jaros R., Bilik P., Ladrova M. (2019). Least Mean Squares and Recursive Least Squares Algorithms for Total Harmonic Distortion Reduction Using Shunt Active Power Filter Control. Energies.

[133] Douglas S. (1994). A Family of Normalized LMS Algorithms. IEEE Signal Process. Lett..

[134] Trilochan P., Mrutyunjaya M., Kumar P.A., Kumar S.S. Sparse LMS Control Algorithm for Fuel Cell Based SAPF. Proceedings of the 2016 IEEE Uttar Pradesh Section International Conference on Electrical, Computer and Electronics Engineering (UPCON).

[135] Martinek R., Bilik P., Baros J., Brablik J., Kahankova R., Jaros R., Danys L., Rzidky J., Wen H. (2020). Design of a Measuring System for Electricity Quality Monitoring within the SMART Street Lighting Test Polygon: Pilot Study on Adaptive Current Control Strategy for Three-Phase Shunt Active Power Filters. Sensors.

[136] Pereira R.R., da Silva C.H., da Silva L.E.B., Lambert-Torres G., Pinto J.O.P. Improving the Convergence Time of Adaptive Notch Filters to Harmonic Detection. Proceedings of the IECON 2010—36th Annual Conference on IEEE Industrial Electronics Society.

[137] Brenna M., Lazaroiu G., Superti-Furga G., Tironi E. (2008). Bidirectional Front End Converter for DG With Disturbance Insensitivity and Islanding-Detection Capability. IEEE Trans. Power Deliv..

[138] Choi J.W., Kim Y.K., Kim H.G. (2006). Digital PLL Control for Single-Phase Photovoltaic System. IEE Proc.-Electr. Power Appl..

[139] Svensson J. (2001). Synchronisation Methods for Grid-Connected Voltage Source Converters. IEE Proc.-Gener. Transm. Distrib..

[140] Chen L.R. (2004). PLL-Based Battery Charge Circuit Topology. IEEE Trans. Ind. Electron..

[141] Karimi-Ghartemani M., Iravani M. (2002). A Nonlinear Adaptive Filter for Online Signal Analysis in Power Systems: Applications. IEEE Trans. Power Deliv..

[142] Karimi-Ghartemani M., Iravani M. (2003). A Signal Processing Module for Power System Applications. IEEE Trans. Power Deliv..

[143] Karimi-Ghartemani M., Iravani M. (2004). A Method for Synchronization of Power Electronic Converters in Polluted and Variable-Frequency Environments. IEEE Trans. Power Syst..

[144] Bojoi R., Griva G., Bostan V., Guerriero M., Farina F., Profumo F. (2005). Current Control Strategy for Power Conditioners Using Sinusoidal Signal Integrators in Synchronous Reference Frame. IEEE Trans. Power Electron..

[145] Yuan X., Allmeling J., Merk W., Stemmler H. Stationary Frame Generalized Integrators for Current Control of Active Power Filters with Zero Steady State Error for Current Harmonics of Concern under Unbalanced and Distorted Operation Conditions. Proceedings of the Conference Record of the 2000 IEEE Industry Applications Conference, Thirty-Fifth IAS Annual Meeting and World Conference on Industrial Applications of Electrical Energy (Cat. No. 00CH37129).

[146] Baros J., Martinek R., Jaros R., Danys L., Soustek L. (2019). Development of Application for Control of SMART Parking Lot. IFAC-PapersOnLine.

[147] Kuncicky R., Kolarik J., Soustek L., Kuncicky L., Martinek R., Zelinka I., Brandstetter P., Trong Dao T., Hoang Duy V., Kim S.B. (2020). IoT Approach to Street Lighting Control Using MQTT Protocol. AETA 2018—Recent Advances in Electrical Engineering and Related Sciences: Theory and Application.

[148] Baros J., Danys L., Jaros R., Martinek R. (2019). Wireless Power Quality Analyser Based on Virtual Instrumentation. IFAC-PapersOnLine.

[149] Danys L., Martinek R., Jaros R., Baros J., Bilik P. (2019). Visible Light Communication System Based on Virtual Instrumentation. IFAC-PapersOnLine.

[150] Martinek R., Danys L., Jaros R. (2020). Adaptive Software Defined Equalization Techniques for Indoor Visible Light Communication. Sensors.

[151] Martinek R., Danys L., Jaros R., Mozny D., Siska P., Latal J. VLC Channel Equalization Simulator Based on LMS Algorithm and Virtual Instrumentation. Proceedings of the 2019 International Symposium on Advanced Electrical and Communication Technologies (ISAECT).

[152] Danys L., Martinek R., Jaros R., Baros J., Bilik P. (2019). OFDM VLC System Based on Virtual Instrumentation and SDR. IFAC-PapersOnLine.

[153] Soustek L., Martinek R., Kuncicky R., Danys L., Baros J. (2019). Possibilities of Intelligent Camera System Based on Virtual Instrumentation: Technology of Broadband LIGHT for “Smart City” Concept. IFAC-PapersOnLine.

